# Nanopore Data-Driven Near-T2T Genome Assembly of *Hippophae rhamnoides* ssp. *mongolica* Rousi and Its Complex Annotation

**DOI:** 10.3390/plants15111726

**Published:** 2026-06-02

**Authors:** Alexander A. Arkhipov, Nadezhda L. Bolsheva, Elena N. Pushkova, Vladislav V. Babenko, Yury A. Zubarev, Valentina A. Krasnova, Vera L. Kovalenko, Fedor D. Kostromskoy, Elizaveta A. Ivankina, Ekaterina M. Dvorianinova, Nikolai M. Barsukov, Daiana A. Krupskaya, Elena V. Borkhert, Ksenia M. Klimina, Alexey A. Dmitriev, Nataliya V. Melnikova

**Affiliations:** 1 Engelhardt Institute of Molecular Biology, Russian Academy of Sciences, 119991 Moscow, Russia; arkhipov.aleksandr2.0@gmail.com (A.A.A.); nlbolsheva@mail.ru (N.L.B.); pushkova18@gmail.com (E.N.P.); lakunina.va@gmail.com (V.A.K.); verakovalenko96@gmail.com (V.L.K.); fkostr@gmail.com (F.D.K.); sigova1567@gmail.com (E.A.I.); eugenevarenyuk@gmail.com (E.M.D.); keepter@yandex.ru (N.M.B.); zhernova.d@ya.ru (D.A.K.); sashai@inbox.ru (E.V.B.); alex_245@mail.ru (A.A.D.); 2 Lopukhin Federal Research and Clinical Center of Physical-Chemical Medicine of Federal Medical Biological Agency, 119435 Moscow, Russia; daniorerio34@gmail.com (V.V.B.); ppp843@yandex.ru (K.M.K.); 3 Federal Altai Scientific Center of Agrobiotechnologies, 656910 Barnaul, Russia; niilisavenko@yandex.ru; 4 Moscow Center for Advanced Studies, 123592 Moscow, Russia

**Keywords:** sea buckthorn, *Hippophae rhamnoides*, nanopore sequencing, T2T genome, genome annotation, FAT, SAD, FAD

## Abstract

Sea buckthorn (*Hippophae rhamnoides* L.) is a valuable plant whose fruits are rich in biologically active compounds. We sequenced the genome of variety Triumf of *H. rhamnoides* ssp. *mongolica* Rousi on the Oxford Nanopore Technologies (ONT) platform. With the Hifiasm algorithm optimized for ONT data, we assembled the 1.17-Gb genome into eleven complete chromosomes and one chromosome consisting of two contigs, which were scaffolded (Chr3). Eleven of twelve chromosomes had pronounced telomeric repeats at both ends and were assembled as telomere-to-telomere (T2T), and one chromosome (Chr12) had telomeric repeats only at one end. We also sequenced transcriptomes of thirteen Triumf organs/tissues and performed genome annotation using these and previously obtained RNA-Seq data for this variety. As a result, we predicted 25,915 genes and 30,527 transcripts. Repetitive elements comprised 66.9% of the genome size. The obtained near-T2T annotated genome assembly of *H. rhamnoides* ssp. *mongolica* variety Triumf enabled the identification of correct composition and sequences of important gene families in sea buckthorn. We demonstrated this with the *FAT*, *SAD*, and *FAD* gene families involved in fatty acid synthesis. Expression analysis revealed which *FAT*, *SAD*, and *FAD* genes are essential for specific organs/tissues. Thus, the Triumf genome assembly is a crucial tool for basic and applied studies of *H. rhamnoides*.

## 1. Introduction

Sea buckthorn (*Hippophae rhamnoides* L.) is a dicotyledonous woody oil plant belonging to the Elaeagnaceae family. It grows in the wild across much of Eurasia, from the Himalayas and Central Asia to Russia and most of Europe [1]. It is cultivated in China, Russia, Romania, Mongolia, and several other countries [1]. Its fruits and leaves contain various biologically active compounds, including essential fatty acids, carotenoids, flavonoids, tocopherols, and vitamins [2,3]. Sea buckthorn extracts exhibit antioxidant, anti-inflammatory, and immunomodulatory properties, as well as protective effects on the nervous and cardiovascular systems [4,5,6,7]. Additionally, sea buckthorn is highly adaptive to adverse environmental conditions, such as saline soils and low temperatures, which makes it a promising candidate for protecting against soil erosion, stabilizing sand dunes, and reclaiming land [1,8,9].

Despite the great potential for using of sea buckthorn fruits in food and pharmaceuticals, modern breeding approaches, such as marker-assisted and genomic selection, are not widely used [1]. This issue is likely due to the insufficient genetic research on this plant. Only a few DNA markers have been proposed for the *Hippophae* species. These markers are predominantly associated with the sex of plants (male or female) and frequently work only on specific populations [10,11,12,13,14,15,16].

Complete and accurate genome assemblies are essential for many plant studies, including transcriptomic analyses, gene family identification, repetitive element representation estimation, valuable trait DNA marker development, genome editing, and pangenome analysis [17,18,19,20]. In recent years, tremendous advances have occurred in third-generation sequencing technologies, providing tools for obtaining continuous chromosome-level plant genome assemblies [21,22,23,24]. These advances have enabled high-quality genome assemblies of *H. rhamnoides* and other *Hippophae* species, predominantly obtained using the Pacific Biosciences (PacBio) platform, with the size from 716 to 1453 Mb [25,26,27,28,29,30,31]. These assemblies are necessary for the molecular genetic studies of sea buckthorn and for using genetic tools to breed this valuable oil crop. However, the progress of the Oxford Nanopore Technologies (ONT) platform in recent years enabled the assembly of telomere-to-telomere (T2T) genomes [32,33,34,35], suggesting that ONT data can be effectively used for genome assembly of sea buckthorn. In addition to genome assembly, most studies require high-quality genome annotation. However, annotation is either unavailable or contains errors for most assembled *H. rhamnoides* genomes [25,26,27]. For example, errors were found in five genes from the *FAT* (acyl-acyl carrier protein (ACP) thioesterase), *SAD* (Stearoyl-ACP Carrier Protein Desaturase), and *FAD* (Fatty Acid Desaturase) families in the *H. rhamnoides* genome assembly under the ID CNA0022752 in the CNGB database (https://db.cngb.org/, Project ID CNP0001846, accessed on 25 November 2025) [36]. Furthermore, *H. rhamnoides* is characterized by high genetic diversity, and the subspecies *H. rhamnoides* ssp. *mongolica* Rousi and *H. rhamnoides* ssp. *sinensis* Rousi, differ greatly [1]. The present study aimed to obtain the T2T genome assembly of *H. rhamnoides* ssp. *mongolica,* the breeding of which focused on developing high-yielding varieties with large high-oil fruits, and to generate high-quality annotation of this assembly.

## 2. Results

### 2.1. De Novo Genome Assembly

DNA sequencing of the variety Triumf of *H. rhamnoides* ssp. *mongolica* on the ONT platform produced 155 Gb of raw reads with an N50 of 31.4 kb. Reads of at least 10 kb with Q10 or higher score (126 Gb in total) were then used for genome assembly with Hifiasm.

The obtained Triumf genome assembly was 1171.9 Mb in length and consisted of 13 contigs. The chromosome number of *H. rhamnoides* is known to be 2n =  24 [37,38]. Therefore, the genome was assembled into eleven complete chromosomes and one chromosome (Chr3) consisting of two contigs. GC content of the Triumf genome assembly was 29.05%. The assembly had a high BUSCO completeness score of 96.8%, with 85.3% of the BUSCOs being complete and single-copy and 11.5% being complete and duplicated.

An initial genome assembly quality assessment based on the available Illumina whole-genome sequencing (WGS) data for Triumf (25.0 Gb, ~10× coverage for a diploid genome; NCBI SRA PRJNA1177110, SRR32309890) showed a QV score of 28.6 and a high estimated completeness of 99.1%, according to Merqury, with a high Illumina alignment rate of 99.3%. However, the low genome coverage likely limited the reliability of the consensus accuracy estimates (QV score). To further evaluate the assembly’s accuracy, we incorporated additional Illumina WGS data from closely related *Hippophae* specimens (SRR32309891 and SRR32309892). Inclusion of the additional data increased the estimated QV score to 45.9, demonstrating the high accuracy of the assembled Triumf genomic sequences. The alignment rates of these data to the Triumf assembly were 99.0% and 98.9%, respectively, indicating minor genomic divergence between the specimens.

### 2.2. Comparison of Hippophae Genome Assemblies

We compared the obtained genome assembly of *H. rhamnoides* ssp. *mongolica* with the available assemblies of the genus *Hippophae*. The Triumf assembly was longer than genome assemblies of most other *Hippophae* species [25,26,27,29,30,31] but shorter than the *H. tibetana* assembly from the study by Wang et al. [28] (Table 1). It is quite possible that there are differences in genome size among different *Hippophae* species. According to the contig N50 parameter, the Triumf assembly was the best among the available genome assemblies of *Hippophae* species (as of 25 November 2025). A comparison of the available sea buckthorn genome assemblies was previously conducted across a wide range of parameters in the study by Chen et al. [31]. However, it is preferable to standardize the methodological approaches used in such an analysis rather than relying on data from individual articles, since the methods for evaluating specific parameters may vary across different articles. For this reason, our study focused on a few significant parameters.

**Figure 1:**
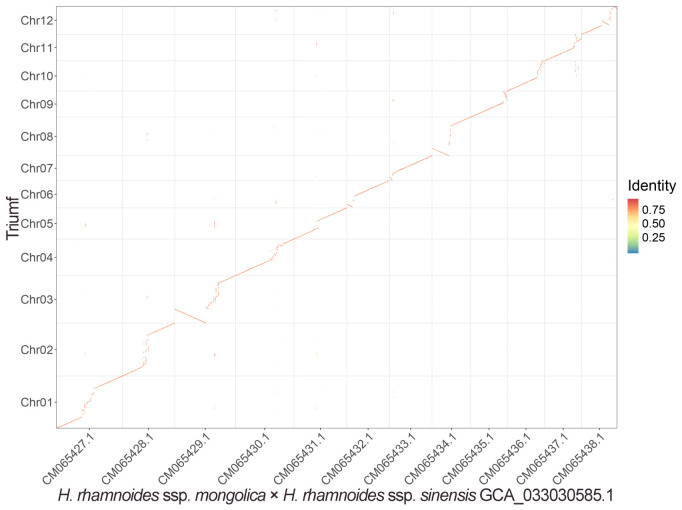
Global alignment of the genome assemblies of *H. rhamnoides* variety Triumf (Y axis) and *H. rhamnoides* ssp. *mongolica* × *H. rhamnoides* ssp. *sinensis* genotype [27], NCBI, GCA_033030585.1 (X axis).

**Figure 2:**
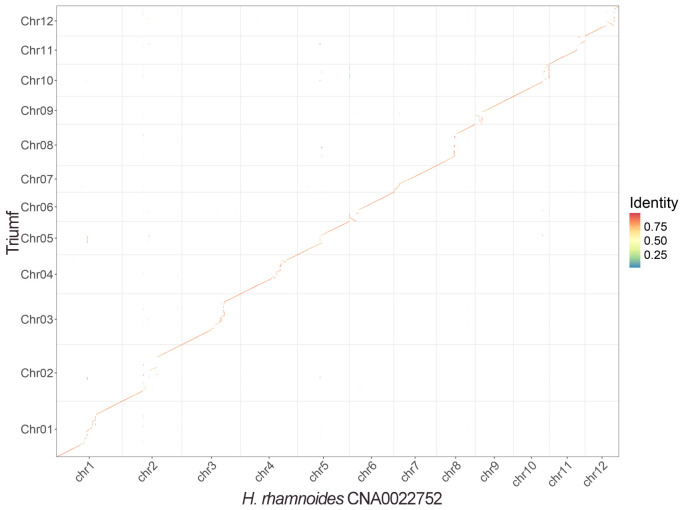
Global alignment of the genome assemblies of *H. rhamnoides* variety Triumf (Y axis) and another *H. rhamnoides* genotype [26], CNGB, CNA0022752 (X axis).

We compared the variety Triumf genome assembly (*H. rhamnoides* ssp. *mongolica*) with the genome assemblies of *H. rhamnoides* ssp. *mongolica* × *H. rhamnoides* ssp. *sinensis* [27] (NCBI, GCA_033030585.1, Figure 1) and *H. rhamnoides* [26] (CNGB, CNA0022752, Figure 2). The *H. rhamnoides* ssp. *mongolica* genome [25] was unavailable for download (http://hipp.shengxin.ren/, accessed on 25 November 2025). The alignment proved that 11 of 12 assembled contigs represented whole chromosomes, and the remaining two contigs were scaffolded in Chr3. The regions of the Triumf genome assembly that made it longer than the other two available *H. rhamnoides* assemblies were predominantly located in the likely centromeric and pericentric regions of chromosomes, where repeated sequences are usually found. The greatest differences in length were characteristic of Chr1, Chr2, Chr3, Chr4, and Chr8.

We also compared the genome assembly of Triumf with those of *H. tibetana* [29] (NCBI, GCA_037013495.1) and *H. gyantsensis* [30] (NCBI, GCA_030763125.1) (Supplementary Figure S1). Similar to the comparison with *H. rhamnoides* genome assemblies, the main differences were present in the likely centromeric and pericentric regions of chromosomes. However, we also observed more noise—mapping of small fragments to other parts of the assemblies. This is likely because of the significant genetic differences between the species being compared.

**Figure 3:**
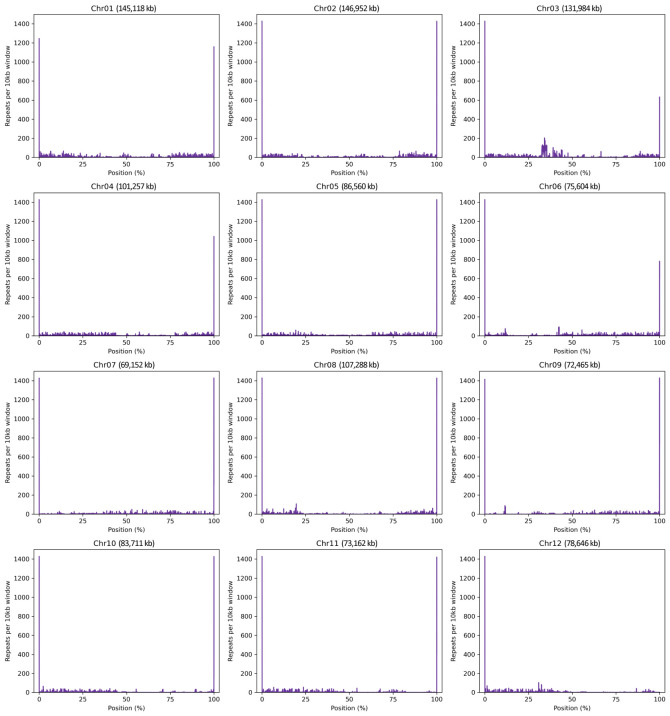
Density of telomeric repeats in the variety Triumf genome assembly. High peaks at both ends of eleven chromosomes and at one end of Chr12 can be seen. Chromosome size is indicated for each chromosome in brackets.

In the Triumf genome assembly, eleven chromosomes had pronounced telomeric repeats at both ends and were assembled as T2T; one chromosome (Chr12) had telomeres only at one end (Figure 3). This result supported the high quality of the Triumf genome assembly. We also analyzed the presence of telomeric repeats in other available sea buckthorn genome assemblies (Supplementary Figure S2). The absence of telomeric repeats at the ends of chromosomes or their mislocation were very common. This may indicate incomplete or erroneous assemblies.

**Figure 4:**
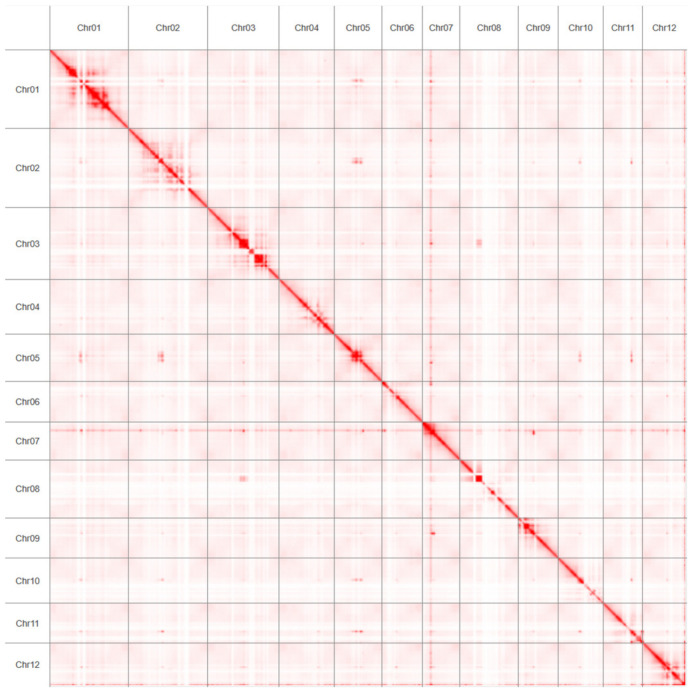
Hi-C interaction heatmap for the variety Triumf genome assembly. The frequencies of Hi-C contacts on chromosomes are presented by colors, varied from white (low) to red (high). All multiple alignments of Hi-C reads were preserved.

We also generated 110.9 Gb of Hi-C genomic data (95× genome coverage) for the variety Triumf and constructed a Hi-C interaction heatmap (Figure 4). The interaction heatmap revealed no significant contacts outside the main diagonal, confirming the accuracy of the obtained genome assembly. Regions with low coverage were associated with low presence of restriction site for the DpnII enzyme (GATC; Supplementary Figure S3), which was used to prepare the Hi-C library.

### 2.3. Annotation of the Variety Triumf Genome Assembly

A total of 2,259,619 repetitive elements were annotated, with a combined length of about 793.5 Mb, accounting for 66.9% of the assembly length. Retrotransposons comprised 41.5% of the length, DNA transposons accounted for 1.6%, and 21.8% of the assembly consisted of unclassified repetitive elements.

Among transposable elements, long terminal repeat elements presented 40.1% of the assembly length, with the Copia and Gypsy repeat families comprising 15.4% and 14.4%, respectively.

**Figure 5:**
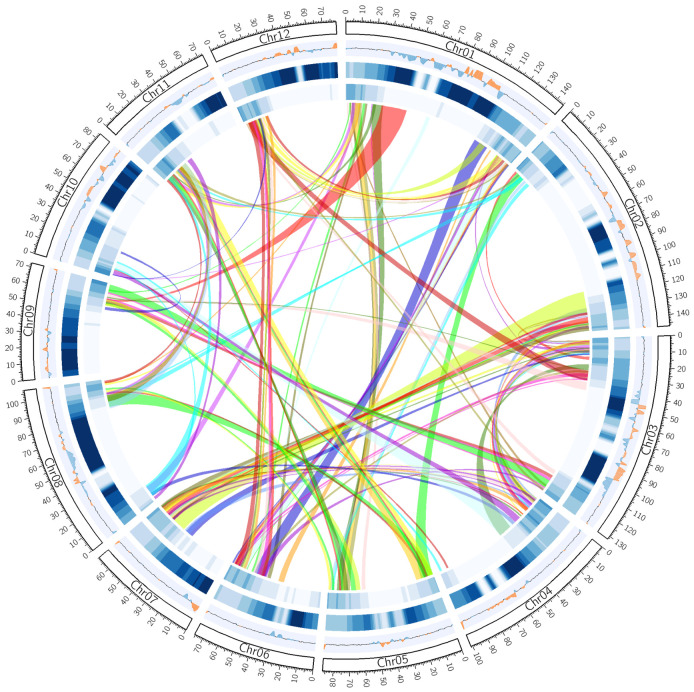
Features of the Triumf genome assembly, shown from the outside inward: chromosome assembly, GC content, repeat density, gene density, and intragenomic synteny (colored lines).

We predicted 25,915 genes and 30,527 transcripts in the obtained genome assembly using transcriptomic data generated in the present study for 13 different organs/tissues of the variety Triumf in two-fold replication (on average, 2.6 million reads per sample): roots, wood, bark, bud scales, early buds, middle buds, late buds, whole leaves, leaf bases, leaf apices, leaf margins, leaf midribs, and pistils. We also used transcriptomic data previously obtained by us for fruit seeds and pulp at four development stages of the same variety (NCBI SRA, PRJNA1163394, SRR35247990–SRR35247993, SRR35247995–SRR35247998) [39]. A Circos plot was drawn based on the GC content, gene and repeat density of the Triumf genome assembly, as well as chromosome synteny (Figure 5).

### 2.4. Identification and Characterization of the FAT Genes of H. rhamnoides

In our previous study, we demonstrated that *FATB* (*Hiprha1gene28610*), *SAD* (*Hiprha1gene18766*), *FAD2* (*Hiprha1gene21624*), *FAD2* (*Hiprha1gene12459*), and *FAD3* (*Hiprha1gene24146*) genes identified using the *H. rhamnoides* CNA0022752 annotated genome assembly (CNGB, Project ID CNP0001846) contained errors [36]. Therefore, we identified genes of the *FAT*, *SAD*, and *FAD* families in the Triumf genome assembly and compared them with genes of these families identified in the CNA0022752 genome assembly.

The AtFATA1 (AT3G25110.1), AtFATA2 (AT4G13050.1), and AtFATB (AT1G08510.1) proteins were initially identified using the TAIR database (https://www.arabidopsis.org/, TAIR, accessed on 25 November 2025) by searching with the “FATA”, “FATB”, “AtFATA”, and “AtFATB” keywords. A further BLASTP search was performed against representative *Arabidopsis thaliana* protein models to identify potential close homologs. A BLASTP search was then performed against the annotated genome assemblies of variety Triumf and genotype CNA0022752. The candidates found were filtered based on minimum identity (30%) to the reference sequences, as well as a set of conserved domains and gene exon-intron structure.

**Figure 6:**
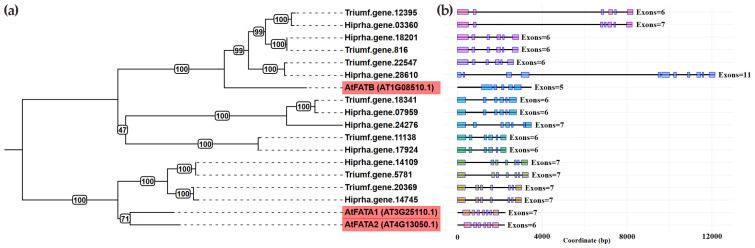
Analysis of the *FATA* and *FATB* genes of *H. rhamnoides.* (**a**) Dendrogram of the FATA and FATB proteins. Constructed using the Neighbor-Joining (NJ) method with a bootstrap value of 1000 and rerooted at midpoint. The proteins of the reference species *A. thaliana* (At…, highlighted in red) and their homologs identified in two annotated genome assemblies of *H. rhamnoides* are shown: Hiprha.gene.xxx—the CNA0022752 genome assembly (CNGB), Triumf.gene.xxx—the Triumf genome assembly obtained in the present study. (**b**) Exon-intron structures of the *FATA* and *FATB* genes. UTRs were not predicted for genes from the *H. rhamnoides* genome assemblies. The *Hiprha.gene.03360*, *Hiprha.gene.28610*, and *Hiprha.gene.24276* genes likely contain errors.

Two *FATA* genes and five *FATB* genes (named *Triumf.gene.xxx*) were identified in the Triumf genome assembly. Two *FATA* genes and six *FATB* genes (named *Hiprha.gene.xxx*) were identified in the CNA0022752 genome assembly. Phylogenetic analysis (Figure 6a) revealed the correspondence of the following genes in the *H. rhamnoides* genome assemblies:

*FATA*:

1. *Triumf.gene.5781—Hiprha.gene.14109*
2. *Triumf.gene.20369—Hiprha.gene.14745*

*FATB:*

1. *Triumf.gene.11138—Hiprha.gene.17924*
2. *Triumf.gene.18341—Hiprha.gene.07959 and Hiprha.gene.24276*
3. *Triumf.gene.22547—Hiprha.gene.28610*
4. *Triumf.gene.816—Hiprha.gene.18201*
5. *Triumf.gene.12395—Hiprha.gene.03360*

Several differences were identified in the *FAT* genes between the two sea buckthorn genome assemblies. For example, the *Hiprha.gene.28610* gene (the CNA0022752 assembly) had 11 exons, though the expected number was 5–6 (Figure 6b). This discrepancy suggested an error in the assembly and subsequent annotation. The corresponding gene in the Triumf genome assembly (*Triumf.gene.22547*) had six exons. Additionally, *Hiprha.gene.03360* (the CNA0022752 assembly) contained seven exons instead of the expected six (Figure 6b). Upon analyzing the conserved domain structure characteristic of this gene family, we discovered that the Acyl-ACP_TE domain was truncated in the region corresponding to the additional exons (Supplementary Figure S4). This suggested a local assembly and annotation error in the CNA0022752 genome assembly. In contrast, the corresponding gene in the Triumf assembly (*Triumf.gene.12395*) contained six exons and a complete set of the expected conserved domains. Furthermore, *Hiprha.gene.24276* (the CNA0022752 assembly) contained seven exons instead of the expected six (Figure 6b). It is likely that a large exon region was split into two separate fragments. Further analysis of this gene domain structure revealed a significantly shortened Acyl-ACP_TE_C domain (Supplementary Figure S4), suggesting an assembly and annotation error. This gene has no complete analog in the Triumf genome assembly; its origin is unclear. It may be a chimera resulting from an assembly error in the CNA0022752 genome. Thus, the Triumf genome assembly enabled correction of the *FAT* gene family sequences.

### 2.5. Identification and Characterization of the SAD Genes of H. rhamnoides

AtSAD1 (AT5G16240.1), AtSAD2 (AT3G02610.2), AtSAD3 (AT5G16230.1), AtSAD4 (AT3G02620.2), AtSAD5 (AT3G02630.1), AtSAD6 (AT1G43800.1), and AtFAB2 (AT2G43710.1) were initially identified using the TAIR database (https://www.arabidopsis.org/, TAIR, accessed on 25 November 2025) using the “stearoyl-ACP desaturase” keyword and a BLASTP search against the representative *A. thaliana* protein models to identify potential closely related genes.

**Figure 7:**
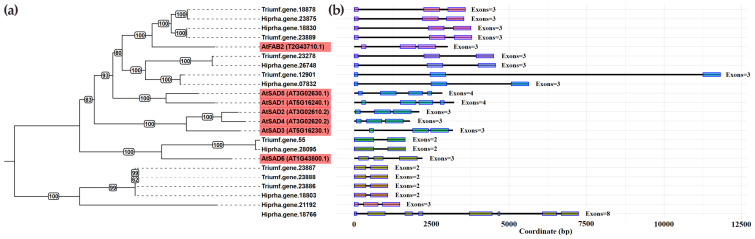
Analysis of the *SAD* genes. (**a**) Dendrogram of the SAD proteins. Constructed using the Neighbor-Joining (NJ) method with a bootstrap value of 1000 and rerooted at midpoint. The proteins of the reference species *A. thaliana* (At…, highlighted in red) and their homologs identified in two annotated genome assemblies of *H. rhamnoides* are shown: Hiprha.gene.xxx—the CNA0022752 genome assembly (CNGB), Triumf.gene.xxx—the Triumf genome assembly obtained in the present study. The chimeric protein Hiprha.gene.18766 was excluded from the dendrogram, as it negatively impacted genomic distance calculations. (**b**) Exon-intron structures of the *SAD* genes. UTRs were not predicted for genes from the *H. rhamnoides* genome assemblies. The *Hiprha.gene.18766* gene likely contains errors.

After filtration, eight *SAD* genes were identified in both the Triumf and CNA0022752 genome assemblies. Phylogenic analysis of the SAD family (Figure 7a) revealed the correspondence of the following genes in the *H. rhamnoides* assemblies:

1. *Triumf.gene.18878—Hiprha.gene.23875*
2. *Triumf.gene.23889—Hiprha.gene.18830*
3. *Triumf.gene.23278—Hiprha.gene.26748*
4. *Triumf.gene.12901—Hiprha.gene.07832*
5. *Triumf.gene.55—Hiprha.gene.28095*
6. *Triumf.gene.23887—Triumf.gene.23888—Triumf.gene.23886—Hiprha.gene.18803—Hiprha.gene.21192—Hiprha.gene.18766*

A number of differences between the *SAD* genes of the annotated *H. rhamnoides* genome assemblies were identified. *Hiprha.gene.18766* contained excessive exons (Figure 7b) and atypical conserved domains (Supplementary Figure S4), resulting from the erroneous merging of two gene models in the CNA0022752 assembly annotation, which led to a chimeric sequence.

### 2.6. Identification and Characterization of the FAD Genes of H. rhamnoides

The AtFAD2 (AT3G12120.1), AtFAD3 (AT2G29980.1), AtFAD4 (AT4G27030.1), AtFAD5 (AT3G15850.1), AtFAD6 (AT4G30950.1), AtFAD7 (AT3G11170.1), and AtFAD8 (AT5G05580.1) were initially identified using the TAIR database (https://www.arabidopsis.org/, TAIR, accessed on 25 November 2025) by searching with the “FAD”, “AtFAD”, and “fatty acid desaturase” keywords. A further BLASTP search was performed against representative *A. thaliana* protein models to identify potential close homologs of these genes.

**Figure 8:**
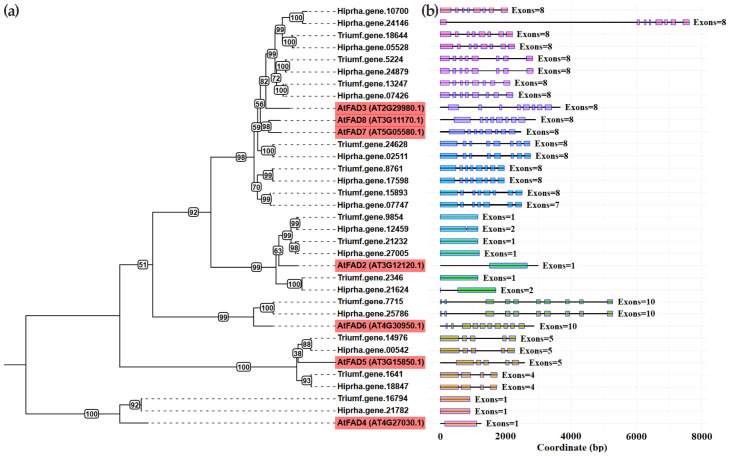
Analysis of the *FAD* genes. (**a**) Dendrogram of the FAD proteins. Constructed using the Neighbor-Joining (NJ) method with a bootstrap value of 1000 and rerooted at midpoint. The proteins of the reference species *A. thaliana* (At…, highlighted in red) and their homologs identified in two annotated genome assemblies of *H. rhamnoides* are shown: Hiprha.gene.xxx—the CNA0022752 genome assembly (CNGB), Triumf.gene.xxx—the Triumf genome assembly obtained in the present study. (**b**) Exon-intron structures of the *FAD* genes. UTRs were not predicted for genes from the *H. rhamnoides* genome assemblies. The *Hiprha.gene.10700*, *Hiprha.gene.24146*, *Hiprha.gene.07747*, *Hiprha.gene.12459*, and *Hiprha.gene.21624* likely contain errors.

After filtration, thirteen *FAD* genes were identified in the Triumf genome assembly and fifteen *FAD* genes in the CNA0022752 genome assembly. Phylogenic analysis of the FAD family (Figure 8a) revealed the correspondence of the following genes in the *H. rhamnoides* assemblies:

*FAD2*:

1. *Triumf.gene.2346—Hiprha.gene.21624*
2. *Triumf.gene.9854—Hiprha.gene.12459*
3. *Triumf.gene.21232—Hiprha.gene.27005*

*FAD3*:

1. *Triumf.gene.13247*—*Hiprha.gene.07426*
2. *Triumf.gene.5224*—*Hiprha.gene.24879*
3. *Triumf.gene.18644*—*Hiprha.gene.05528*
4. *Hiprha.gene.10700* and *Hiprha.gene.24146* did not have equivalents in the Triumf genome assembly

*FAD4*:

1. *Triumf.gene.16794*—*Hiprha.gene.21782*

*FAD5*:

1. *Triumf.gene.14976*—*Hiprha.gene.00542*
2. *Triumf.gene.1641*—*Hiprha.gene.18847*

*FAD6*:

1. *Triumf.gene.7715*—*Hiprha.gene.25786*

*FAD7/8*:

1. *Triumf.gene.24628*—*Hiprha.gene.02511*
2. *Triumf.gene.15893*—*Hiprha.gene.07747*
3. *Triumf.gene.8761*—*Hiprha.gene.17598*

A number of differences in the *FAD* family genes between the annotated *H. rhamnoides* genome assemblies were also identified. In the Triumf assembly, only three *FAD3* genes were identified, compared to five in the CNA0022752 genome assembly. The additional genes, *Hiprha.gene.10700* and *Hiprha.gene.24146*, contained truncated conserved domain structures (Supplementary Figure S4). Furthermore, *Hiprha.gene.24146* contained an atypically long intron sequence (Figure 8b), which may have resulted from either an assembly or annotation error.

*Hiprha.gene.07747* contained only seven exons instead of the expected eight (Figure 8b). Further inspection of the alignments of obtained RNA-Seq reads determined this to be an annotation error in the CNA0022752 genome assembly (Supplementary Figure S5). Both seven-exon and eight-exon variants of the corresponding *Triumf.gene.15893* gene are present in the Triumf annotation.

A single exon of *Hiprha.gene.12459* was incorrectly split into two parts, while *Hiprha.gene.21624* contained a short additional exon sequence (Figure 8b). These exon-intron structures were not supported by the RNA-Seq read alignment analysis and therefore attributed to annotation errors (Supplementary Figures S6 and S7).

The location of *FAT*, *SAD*, and *FAD* genes on twelve chromosomes of the Triumf genome assembly is presented in Supplementary Figure S8.

### 2.7. Expression Analysis of FAT, SAD, and FAD Genes

We also analyzed expression levels of the identified *FAT*, *SAD*, and *FAD* family genes in 34 samples of sea buckthorn variety Triumf as read counts per million reads (CPM), calculated based on the transcriptomic data obtained in this study for 13 different organs/tissues (roots, wood, bark, buds, bud scales, leaves, and pistils), as well as our previously obtained transcriptomic data from the NCBI database (PRJNA1163394) for fruit seeds and pulp (Supplementary Table S1). Certain genes exhibited the highest levels of expression in specific organs/tissues at particular stages of development. For instance, *FATA* (*Triumf.gene.5781* and *Triumf.gene.20369*), *SAD* (*Triumf.gene.18878*, *Triumf.gene.23889*, *Triumf.gene.23278*, *Triumf.gene.12901*, and *Triumf.gene.55*), and *FAD2* (*Triumf.gene.2346*) had high expression levels in seeds and pulp of sea buckthorn fruits. These genes are likely involved in the synthesis of fatty acids in the fruits. Some genes, including *FATB* (*Triumf.gene.12395*), exhibited moderate expression in all of the studied organs/tissues. These genes are likely to be involved in processes occurring in most sea buckthorn plant cells. We also identified genes that were virtually unexpressed in the studied samples, including *FATB* (*Triumf.gene.18341*) and *SAD* (*Triumf.gene.23888*, *Triumf.gene.23887*, and *Triumf.gene.23886*). These genes may only function under specific conditions, such as stress. Alternatively, they may not be expressed at all in sea buckthorn plants. This issue requires further study.

## 3. Discussion

We obtained a near-T2T genome assembly of the variety Triumf of *H. rhamnoides* ssp. *mongolica* using ONT sequencing and the Hifiasm genome assembler optimized for ONT data. The assembly consisted of 13 contigs—eleven complete chromosomes and one chromosome in two parts, which were then scaffolded.

The Triumf genome assembly was longer than previous *H. rhamnoides* assemblies [25,26,27]: 1171.9 Mb versus 730.5, 752.1, and 849.0 Mb. Comparison of the genome assembly of Triumf with those of other available *Hippophae* species revealed that the main differences were associated with the centromeric and pericentric regions of chromosomes. The PacBio reads, predominantly used to assemble the *Hippophae* genomes [25,26,28], were shorter than the ONT reads [40]. The shorter read length could result in problems when assembling extended genomic regions rich in repetitive sequences [41]. Therefore, the centromeric and pericentric regions, which contain repeats [42,43], may be underrepresented in *Hippophae* genomes initially assembled using only PacBio reads and then scaffolded with Hi-C data. To assemble the *Hippophae* genomes from ONT reads, NextPolish [30] and NextDenovo [27] algorithms were used. However, as of 25 August 2025, ONT recommends using the Hifiasm (ONT) algorithm to obtain near-T2T genome assemblies from standard ONT Simplex reads based on a recent study [32] (https://nanoporetech.com/news/breakthrough-algorithm-enables-partially-phased-near-telomere-to-telomere-assembly-using-standard-oxford-nanopore-simplex-reads, accessed on 25 November 2025). For the variety Triumf genome assembly, we used Hifiasm (ONT) and obtained excellent results. Furthermore, we generated ONT reads with a high N50 parameter (31.4 kb), and a significant proportion of reads exceeded 50 kb. This could positively impact the quality of the obtained assembly. Therefore, the variety Triumf assembly may contain repeated regions of the genome that were not assembled in previous *H. rhamnoides* studies, surpassing previous assemblies in completeness. We also obtained Hi-C reads, and the contact map confirmed the accuracy of the Triumf genome assembly.

The analysis of telomeric repeats, which are located at the ends of chromosomes, also supported the high quality of the variety Triumf genome assembly. Eleven of the twelve chromosomes had telomeres at both ends, and one chromosome had a telomere at one end. In contrast, analysis of other *Hippophae* genome assemblies revealed errors in the location of telomeric repeats (i.e., within the chromosomes rather than at their ends) or their near absence. This discrepancy may be because of the initial incompleteness of the genome assemblies followed by merging contigs into scaffolds using Hi-C data.

Analyzing the *FAT*, *SAD*, and *FAD* gene families in the annotated Triumf genome assembly allowed us to obtain the correct sequences of genes of these families, which had errors in the annotated CNA0022752 genome assembly, the only available annotated genome assembly of *H. rhamnoides* as of 25 November 2025 (CNGB, Project ID CNP0001846). The *FAT* family analysis suggested that the *Hiprha.gene.24276* gene in the CNA0022752 assembly is potentially a chimera gene that is actually absent. Among the *SAD* genes, *Hiprha.gene.18766* in the CNA0022752 assembly had an atypical structure that probably resulted from the erroneous merging of two gene models. This problem was not presented in the Triumf genome assembly. A significant number of errors were revealed in the *FAD* genes of the CNA0022752 assembly. There were wrong additional *FAD* genes with truncated conserved domains. Additionally, lack of exons, incorrect additional exons, and erroneous split of one exon into two parts were revealed for *FAD* genes in the CNA0022752 annotation compared to the Triumf annotation and confirmed by the analysis of transcriptomic data. To annotate the Triumf genome assembly, we used a representative set of transcriptomic data, including data obtained for fruits in which fatty acid synthesis actively occurred. To annotate the CNA0022752 assembly, fewer transcriptomic samples were used, and no fruits were analyzed that could decrease the annotation results for the *FAT*, *SAD*, and *FAD* genes. Additionally, other tools were used to annotate the CNA0022752 assembly in comparison with the Triumf assembly. This may have also contributed to the differences in the annotations. *FAT* genes play an essential role in fatty acid biosynthesis by hydrolyzing acyl-ACP complexes to release free fatty acids in plastids [44]. *SAD* genes are essential for converting of saturated fatty acids into monounsaturated ones [45], while *FAD* genes play a key role in the synthesis of polyunsaturated fatty acids [46,47]. To comprehend the process of fatty acid biosynthesis in sea buckthorn fruits during ripening, it is essential to determine the precise sequences of the *FAT*, *SAD*, and *FAD* genes.

T2T genomes are becoming the new standard for genome assemblies, not only for model organisms but also for a wide range of plant species. T2T genomes provide insights into genome organization and function, distribution of repetitive elements, polyploid features and evolution, genetic diversity, and host–pathogen interactions [18,48,49,50,51,52,53,54,55].

We obtained a near-T2T genome of *H. rhamnoides* ssp. *mongolica* variety Triumf using only ONT sequencing data. It became possible due to significant advancements in ONT technology and genome assembly tools. As of 20 April 2026, the Triumf assembly is a reference for *H. rhamnoides* in the NCBI Genome database (GCA_056149425.1). The variety Triumf was created as a part of the Russian breeding program and differs from the *H. rhamnoides* genotypes used in previous genome assembly studies. The Triumf genome can be useful for identifying differences between genetically distant genotypes at the whole-genome level. Furthermore, Triumf is a high-quality fruit variety, and its genome can be valuable for identifying gene variants that determine important characteristics of sea buckthorn fruits for food and pharmaceutical applications. The variety Triumf genome also provides a solid foundation for studying particular gene families, including their representation and structure. It is an essential tool for various basic and applied studies, as well as genome editing of *H. rhamnoides*.

## 4. Materials and Methods

### 4.1. DNA Extraction and ONT Sequencing

The variety Triumf was selected for the study. It is characterized by high yields and large, cylindrical, red fruits with dense flesh, firm skin, and high carotenoid content.

Nuclei isolation was performed using the LN2 plant tissue protocol (https://www.pacb.com/wp-content/uploads/Procedure-checklist-Isolating-nuclei-from-plant-tissue-using-LN2-disruption.pdf, accessed on 25 November 2025) with some modifications. Young sea buckthorn leaves weighing 0.5 g were frozen in liquid nitrogen and ground in a mortar for 20 min. The resulting powder was transferred to 25 mL of ice-cold NIB buffer consisting of 1× HB buffer (10 mM Tris pH 9.4 (Diaem, Moscow, Russia), 80 mM KCl (Helicon, Moscow, Russia), and 10 mM EDTA (Diaem)), supplemented with 0.5 M sucrose (Merck, Rahway, NJ, USA), 1% Triton X-100 (Diaem), 1% β-mercaptoethanol (NeoFroxx, Einhausen, Germany), 2% PVP K30 (CDH, New Delhi, India), and 100 mM spermine and spermidine (Sigma-Aldrich, St. Louis, MO, USA). The suspension was placed on ice and stirred on a rocker at 100 rpm for 15–20 min. Then, it was centrifuged at 3000× *g* and 4 °C for 20 min. The supernatant was carefully removed, and the precipitate was resuspended in 4 mL of a 36% iodixanol solution (Sigma-Aldrich) in 1× HB buffer. The solution was then centrifuged for 30 min at 5000× *g* and 4 °C. The upper phase was transferred to a new tube and resuspended in 12.5 mL of NIB buffer. The tube was then centrifuged for 10 min at 3000× *g* and 4 °C. The supernatant was then removed. Washing in NIB buffer was repeated one more time. The resulting precipitate was washed with 1 mL of HB buffer and centrifuged for 10 min at 3000× *g* and 4 °C.

To lyse the nuclei, the precipitate was transferred to 1 mL of Carlson buffer prewarmed to 65 °C containing 100 mM Tris-HCl, pH 9.5 (Diaem), 2% CTAB (Helicon), 1.4 M NaCl (Diaem), 1% PEG-8000 (PanReac AppliChem, Darmstadt, Germany), 20 mM EDTA (Diaem), 3 μL β-mercaptoethanol (NeoFroxx), 1% PVP K30 (CDH), and 4 μL RNase A (100 mg/mL; 7000 units/mL; Qiagen, Chatsworth, MD, USA). The mixture was incubated at 65 °C for one hour then cooled to room temperature. Then, an equal volume of chloroform (Diaem) was added to the homogenate. The tube was gently inverted ten times until a homogeneous emulsion formed. The mixture was then centrifuged for 5 min at 3000× *g* and 4 °C. The aqueous phase was carefully transferred to a new tube, and the chloroform extraction procedure was repeated.

DNA was precipitated from the aqueous phase with 0.7 volumes of isopropanol (VWR Chemicals, Radnor, PA, USA). The tube was then centrifuged for 5 min at 3000× *g* and 4 °C. The supernatant was subsequently removed. The DNA was air-dried for 2 min, dissolved in 2 mL of G-buffer (Blood & Cell Culture DNA Mini Kit, Qiagen), and 25 μL of proteinase K (>600 mU/mL; Qiagen) was added. The mixture was incubated at 56 °C overnight. Then, the DNA was purified using Blood & Cell Culture DNA Mini Kit columns (Qiagen) according to the manufacturer’s protocol.

The quality and concentration of the DNA were assessed using a NanoDrop 2000C spectrophotometer (Thermo Fisher Scientific, Waltham, MA, USA) and a Qubit 4.0 fluorometer (Thermo Fisher Scientific). Similarity of the DNA concentration values obtained by the two methods served as an additional criterion for sample purity. The sizes of the isolated DNA fragments were evaluated by electrophoresis in a 0.8% agarose gel.

The SQK-LSK114 kit (Oxford Nanopore Technologies, Oxford, UK) was used to prepare the DNA library for ONT sequencing. Genome sequencing was performed using a PromethION (Oxford Nanopore Technologies) with two R10.4.1 flow cells (Oxford Nanopore Technologies).

### 4.2. Construction and Sequencing of Hi-C Library

Vacuum infiltration with formaldehyde was used to fix the chromatin. Next, nuclei were isolated from the fixed leaves as described above. At the final stage, the resulting precipitate was washed with PBS. According to the optimized protocol [56], the following steps were performed: restriction with DpnII (New England Biolabs, Ipswich, MA, USA); biotin labeling with Biotin-15-dCTP (Biosan, Novosibirsk, Russia); and subsequent ligation of DNA fragments with T4 DNA ligase (New England Biolabs). The DNA was then isolated using phenol-chloroform extraction. Additionally, DNA purification was performed using MagicPure DNA beads (TransGen, Beijing, China). Biotin was then removed from the unligated ends of the DNA fragments. The Hi-C library was prepared using the Qiaseq FX DNA kit (Qiagen) up to the amplification step. Next, Streptavidin Magnetic Beads (New England Biolabs) were used to enrich the biotinylated fragments. Finally, the obtained Hi-C library was amplified. The quality of the library was assessed using a 2100 Bioanalyzer (Agilent Technologies, Santa Clara, CA, USA), and the concentration was measured with a Qubit 4.0 fluorometer (Thermo Fisher Scientific). Sequencing was performed on a NovaSeq X Plus instrument (Illumina, San Diego, CA, USA) with 150 + 150 nucleotide reads.

### 4.3. Transcriptome Sequencing

For the transcriptomic analysis, plant material was collected for the sea buckthorn variety Triumf. The cuttings, which were collected at the Federal Altai Scientific Center of Agrobiotechnologies (Barnaul, Russia), were cut into pieces that were 13–15 cm long and placed in containers with an indolebutyric acid solution. After 24 h, the cuttings were transferred to containers with water. The water was changed daily, and samples were taken at necessary points as the plants grew. The samples included roots, wood, bark, bud scales (from dormant buds), early buds (beginning of swelling), middle buds (swollen/unfolding buds), and late buds (fully opened buds), leaves (whole and separated into bases, apices, margins, and midribs), and pistils. The plant material was collected in 1.5 mL tubes (at least three samples for each organ/tissue), immediately frozen in liquid nitrogen, and stored in a freezer at −70 °C until RNA extraction. Thirteen pools of samples were subjected to further transcriptomic studies in twofold replicates.

Before RNA extraction, the samples were ground in 1.5 mL tubes in liquid nitrogen using disposable homogenization pestles attached to a DCD701D2 cordless drill/driver (DeWALT, Towson, MD, USA) to a fine powder to prevent thawing. RNA extraction was performed based on a modified CTAB buffer protocol [57]. Total RNA was then purified using a CleanRNA kit (Evrogen, Moscow, Russia), following the manufacturer’s protocol and including a DNase I treatment step. RNA quality and concentration were assessed using horizontal agarose gel electrophoresis and a Qubit 4.0 fluorometer (Thermo Fisher Scientific).

To prepare the cDNA libraries, we used the MagicPure mRNA Kit (TransGen), TransNGS Fast RNA-Seq Library Prep Kit for Illumina (TransGen), and MagicPure Size Selection DNA Beads (TransGen) in accordance with the manufacturer’s protocols. We assessed the quality of the resulting cDNA libraries using the Qsep1-Plus capillary electrophoresis system (BiOptic, New Taipei City, Taiwan) and horizontal electrophoresis in a 2% agarose gel. The concentration of the libraries was determined using a Qubit 4.0 fluorometer (Thermo Fisher Scientific). The cDNA libraries were sequenced on NextSeq2000 (Illumina) with 100 + 100 nucleotide reads.

### 4.4. Genome Assembly and Evaluation

Basecalling of ONT data was performed using the Dorado software package (dorado basecaller v.1.0.2, https://github.com/nanoporetech/dorado, accessed on 25 November 2025) with a minimum average read quality of Q10 (--min-qscore 10). The most accurate model, dna_r10.4.1_e8.2_400bps_sup@v5.2.0 (sup), was used for this purpose. The length and quality of the resulting ONT reads were assessed via NanoPlot (v.1.44.1) [58].

The genome assembly was performed using the Hifiasm algorithm (v.0.25.0) [32] with a parameter set optimized for ONT reads (--ont). The ONT reads were filtered to a minimum length of 10 kb. The following assembly parameters were used: --telo-m CCCTAAA.

Basic assembly parameters, such as length, number of contigs, etc., were obtained and visualized using QUAST (v.5.3.0) [59]. BUSCO (v.5.8.3) [60] was used to evaluate the completeness of the genome assembly using the eudicots_odb10 database of universal single-copy orthologs. BUSCO was used in the eukaryotic genomic data analysis mode (--genome).

Global alignment of the genome assemblies was performed using LAST (v.1639) [61]. Only contigs 10 Mb or longer were considered for alignment. The --uRY128 option, which was designed for whole-genome comparisons, was used for alignment; initial matches between query and reference sequences were searched at ~1/128 of positions in each sequence. Global alignment of the genome assemblies was also performed using minimap2 (v2.3.1) (https://github.com/lh3/minimap2, accessed on 20 April 2026). Only contigs 10 Mb or longer were considered for alignment. The asm10 alignment preset was used. The alignments were visualized with paf2dotplot (https://github.com/moold/paf2dotplot, accessed on 20 April 2026).

The TIDK software package (v.0.2.64) [62] was used to detect telomeric sequences in the assemblies. The CCCTAAA telomeric motif, characteristic of sea buckthorn, was searched in 10 kb windows. Telomere repeat density per 10 kb windows was visualized with matplotlib (v3.10.8) [63].

Hi-C reads were aligned to the obtained Triumf genome assembly using HiC-Pro v3.1.0 [64]. The mapping quality filter was disabled, and all multi-mapped reads were kept. The contact map was generated with Juicer Tools (v.1.22.01) and visualized with Juicebox (v.1.11.08) [65].

DpnII restriction site (GATC) density across the Triumf assembly was computed in 50 kb windows, normalized to sites per kilobase, smoothed using a centered rolling mean (5 bins), and visualized with matplotlib (v3.10.8) [63].

Both the genomic and transcriptomic Illumina reads were trimmed and filtered with fastp (v.1.0.1) using default parameters [66].

Illumina genome sequencing data (NCBI SRA, PRJNA1177110, obtained for varieties Triumf (SRR32309890), 681-09-01 (SRR32309891), and Zarnitsa (SRR32309892)) were used to validate the assembly quality with the following approaches: read mapping using bowtie2 (v2.5.5) [67] and k-mer distribution analysis with Jellyfish (v.2.3.1) [68] with k set to 20. For the k-mer analysis, the high count of the histogram was set at 1,000,000 as recommended for highly repetitive genomes. Genome size and duplication rate estimation was performed with GenomeScope2 (v.2.0.1) [69].

QV score and completeness estimation was calculated using Meryl (v1.4.1) and Merqury (v1.3) [70] with k set to 20.

### 4.5. Genome Annotation

Repetitive genomic region annotation and genome masking were performed with RepeatModeler 2.0.7 and RepeatMasker 4.2.1 [71]. RepeatModeler 2.0.7 was used with the -LTRStruct option enabling the LTR structural discovery pipeline and databases dfam 3.9 [72] and Repbase 2018 [73]. Structural annotation of the assembly was performed with the BRAKER3 (v.3.0.8) [74] using the Viridiplantae protein database [75] and Illumina transcriptome sequencing data obtained for variety Triumf at the present study and previously (NCBI SRA, PRJNA1163394).

GC content, intragenomic synteny, and genome coverage of gene and repeat annotations were calculated per 250 kb windows and visualized with Circos (v0.69.0) [76].

Intragenomic synteny was calculated with MCScanX (v1.0.0) [77], based on BLASTP (v2.17.0) self-alignment of protein sequences derived from primary transcripts. Only the top five best hits for each query were kept. For collinearity detection, the minimum number of genes required to call a collinear block was set to 15, with gap penalty of -5 and max gaps of 10.

### 4.6. Gene Identification

Representative FAT, SAD, and FAD protein models were retrieved from The Arabidopsis Information Resource (TAIR; https://www.arabidopsis.org/, accessed on 25 November 2025) and used as queries for a local BLASTP (v2.17.0) search against *H. rhamnoides* genome assemblies of variety Triumf and genotype CNA0022752 (https://db.cngb.org/, Project ID CNP0001846, accessed on 25 November 2025) [26]. Candidate *H. rhamnoides* protein sequences with less than 30% sequence identity to the reference proteins were discarded.

Protein sequences from *A. thaliana* and *H. rhamnoides* were further analyzed using a local HMMER 3.0 (http://hmmer.org/, accessed on 25 November 2025) search against the Pfam conserved domain database [78]. *H. rhamnoides* sequences lacking the characteristic conserved domains of FAT, SAD, and FAD families were excluded.

Exon-intron structures of the candidate genes were visualized using the ggplot2 v.4.0.1 R package [79] and manually inspected. The retained protein sequences were aligned using MAFFT v7.525. Phylogenetic trees were constructed using RapidNJ v2.3.2 with 1000 bootstrap replicates and visualized with iTOL v7.3 [80]. Gene locations on the chromosome assembly were visualized with Circos (v0.69.0) [76].

To further assess the differences between the relevant gene models, the transcriptomic Illumina reads obtained in the present study and earlier (NCBI SRA, PRJNA1163394) were aligned to the annotated *H. rhamnoides* genome assemblies using PPline [81]. The resulting alignments were visualized with IGV (v2.19.2) [82]. Gene expression analysis was performed using PPline.

## Figures and Tables

**Table 1:** Statistics of genome assemblies of the variety Triumf and other *Hippophae* genotypes.

Species, Reference, Assembly Project	Method	Length, Mb	**Contig N50, Mb**
*H. rhamnoides* ssp. *mongolica* (variety Triumf), present study	ONT, Hi-C	1171.9	83.7
*H. rhamnoides*, Ref. [26], CNGB CNP0001846	PacBio, Hi-C	730.5	3.5
*H. rhamnoides* ssp. *mongolica × H. rhamnoides* ssp. *sinensis*, Ref. [27], NCBI PRJNA1003561	ONT, Hi-C	918.6 (752.1) ^1^	14.8
*H. rhamnoides* ssp. *mongolica*, Ref. [25] ^2^	PacBio, Hi-C	849.0	2.2
*H. tibetana*, Ref. [29], NCBI PRJNA1070417	PacBio, Hi-C	957.2	36.4
*H. tibetana*, Ref. [28], NCBI PRJNA796061, CNGB CNP0003543	PacBio, Hi-C	1452.8	74.3
*H. gyantsensis*, Ref. [30], NCBI PRJNA997223	ONT, Hi-C	716.3	23.2
*H. neurocarpa*, Ref. [31]	PacBio, Hi-C	1002.5	24.4

^1^ based on the article data [27] and, in brackets, on the NCBI genome assembly GCA_033030585.1 (obtained in the study [27]); ^2^ the genome assembly is unavailable (http://hipp.shengxin.ren/, accessed on 25 November 2025).

## Data Availability

The data generated in this study are available at NCBI under the BioProject accession numbers PRJNA1177110 (genomic data, linked to BioSample SAMN46736376) and PRJNA1163394 (transcriptomic data, linked to BioSamples SAMN60524364–SAMN60524389). Triumf genome assembly annotation is available at Zenodo (https://zenodo.org/records/19655687, accessed on 20 April 2026).

## Supplementary Materials

The following supporting information can be downloaded at: https://www.mdpi.com/article/10.3390/plants15111726/s1, Figure S1: Global alignment of the genome assemblies of *H. rhamnoides* variety Triumf and other *Hippophae* species; Figure S2: Density of telomeric repeats in the analyzed genome assemblies of *Hippophae* species; Figure S3: Density of restriction site for the DpnII enzyme (GATC) in the *H. rhamnoides* variety Triumf genome assembly; Figure S4: Conserved domain structure of the FAT, SAD, and FAD proteins identified in the *H. rhamnoides* genome assemblies of variety Triumf and genotype CNA0022752; Figure S5: Mapping of RNA-Seq reads to the *H. rhamnoides* Triumf and CNA0022752 (CNGB, Project ID CNP0001846) genome assemblies for the *FAD7/8* gene region: (a) *Triumf.gene.15893* in the Triumf assembly and (b) *Hiprha.gene.07747* in the CNA0022752 assembly; Figure S6: Mapping of RNA-Seq reads to the *H. rhamnoides* Triumf and CNA0022752 (CNGB, Project ID CNP0001846) genome assemblies for the *FAD2* gene region: (a) *Triumf.gene.9854* in the Triumf assembly and (b) *Hiprha.gene.12459* in the CNA0022752 assembly; Figure S7: Mapping of RNA-Seq reads to the *H. rhamnoides* Triumf and CNA0022752 (CNGB, Project ID CNP0001846) genome assemblies for the *FAD2* gene region: (a) *Triumf.gene.2346* in the Triumf assembly and (b) *Hiprha.gene.21624* in the CNA0022752 assembly; Figure S8: Location of identified in the present study *FAT*, *SAD*, and *FAD* genes on twelve chromosomes of the sea buckthorn variety Triumf genome assembly; Table S1. Expression levels of *FAT*, *SAD*, and *FAD* genes in 34 sea buckthorn Triumf samples calculated as CPM (counts per million reads) based on transcriptomic data obtained in the present study for 13 different organs/tissues (roots, wood, bark, buds, bud scales, leaves, and pistils) and from the NCBI BioProject PRJNA1163394 (fruit seeds and pulp).

## References

[1] Nybom H., Ruan C., Rumpunen K. (2023). The Systematics, Reproductive biology, biochemistry, and breeding of sea buckthorn-A review. Genes.

[2] Ciesarova Z., Murkovic M., Cejpek K., Kreps F., Tobolkova B., Koplik R., Belajova E., Kukurova K., Dasko L., Panovska Z. (2020). Why is sea buckthorn (*Hippophae rhamnoides* L.) so exceptional? A review. Food Res. Int..

[3] Jasniewska A., Diowksz A. (2021). Wide spectrum of active compounds in sea buckthorn (*Hippophae rhamnoides*) for disease prevention and food production. Antioxidants.

[4] Du W., Ding J., Lu S., Wen X., Hu J., Ruan C. (2022). Identification of the key flavonoid and lipid synthesis proteins in the pulp of two sea buckthorn cultivars at different developmental stages. BMC Plant Biol..

[5] Wang Z., Zhao F., Wei P., Chai X., Hou G., Meng Q. (2022). Phytochemistry, health benefits, and food applications of sea buckthorn (*Hippophae rhamnoides* L.): A comprehensive review. Front. Nutr..

[6] Chen Y., He W., Cao H., Wang Z., Liu J., Wang B., Wang C. (2024). Research progress of sea buckthorn (*Hippophae rhamnoides* L.) in prevention and treatment of cardiovascular disease. Front. Cardiovasc. Med..

[7] Gatlan A.M., Gutt G. (2021). Sea buckthorn in plant based diets. An analytical approach of sea buckthorn fruits composition: Nutritional value, applications, and health benefits. Int. J. Environ. Res. Public Health.

[8] Stobdan T., Dolkar P., Chaurasia O., Kumar B. (2017). Seabuckthorn (*Hippophae rhamnoides* L.) in trans-Himalayan Ladakh, India. Def. Life Sci. J..

[9] Ma Q.G., He N.X., Huang H.L., Fu X.M., Zhang Z.L., Shu J.C., Wang Q.Y., Chen J., Wu G., Zhu M.N. (2023). *Hippophae rhamnoides* L.: A comprehensive review on the botany, traditional uses, phytonutrients, health benefits, quality markers, and applications. J. Agric. Food Chem..

[10] Zeng Z., Wang J., Tian Z., Norbu N., Chen Y., Chen J., Zhang W., Qiong L. (2024). Development of sex-specific molecular markers for early sex identification in *Hippophae gyantsensis* based on whole-genome resequencing. BMC Plant Biol..

[11] Zeng Z., Wang R., Wang J., Chen Y., Wang Y., Song Z., Zhang W., Qiong L. (2024). Development and validation of sex-linked molecular markers for rapid and accurate identification of male and female *Hippophae tibetana* plants. Sci. Rep..

[12] Ding J., Ruan C.J., Guan Y., Shan J.Y., Li H., Bao Y.H. (2016). Characterization and identification of ISSR markers associated with oil content in sea buckthorn berries. Genet. Mol. Res. GMR.

[13] Korekar G., Sharma R.K., Kumar R., Meenu, Bisht N.C., Srivastava R.B., Ahuja P.S., Stobdan T. (2012). Identification and validation of sex-linked SCAR markers in dioecious *Hippophae rhamnoides* L. (Elaeagnaceae). Biotechnol. Lett..

[14] Zeng Z., Zhang S., Tan X., Tso N., Shang Z., Zhang J., Li W., Wang J., Zhang W., Qiong L. (2025). Development and application of sex-specific indel markers for *Hippophae salicifolia* based on third-generation sequencing. BMC Plant Biol..

[15] Zhou W., Wang Y., Zhang G., Luan G., Chen S., Meng J., Wang H., Hu N., Suo Y. (2018). Molecular sex identification in dioecious *Hippophae rhamnoides* L. via RAPD and SCAR markers. Molecules.

[16] Li H., Ruan C.J., Teixeira da Silva J.A., Liu B.Q. (2010). Associations of SRAP markers with dried-shrink disease resistance in a germplasm collection of sea buckthorn (*Hippophae* L.). Genome.

[17] Bernal-Gallardo J.J., de Folter S. (2024). Plant genome information facilitates plant functional genomics. Planta.

[18] Garg V., Bohra A., Mascher M., Spannagl M., Xu X., Bevan M.W., Bennetzen J.L., Varshney R.K. (2024). Unlocking plant genetics with telomere-to-telomere genome assemblies. Nat. Genet..

[19] Gladman N., Goodwin S., Chougule K., Richard McCombie W., Ware D. (2023). Era of gapless plant genomes: Innovations in sequencing and mapping technologies revolutionize genomics and breeding. Curr. Opin. Biotechnol..

[20] Jayakodi M., Shim H., Mascher M. (2025). What are we learning from plant pangenomes?. Annu. Rev. Plant Biol..

[21] Medhi U., Chaliha C., Singh A., Nath B.K., Kalita E. (2025). Third generation sequencing transforming plant genome research: Current trends and challenges. Gene.

[22] Pucker B., Irisarri I., de Vries J., Xu B. (2022). Plant genome sequence assembly in the era of long reads: Progress, challenges and future directions. Quant. Plant Biol..

[23] Sun Y., Shang L., Zhu Q.H., Fan L., Guo L. (2022). Twenty years of plant genome sequencing: Achievements and challenges. Trends Plant Sci..

[24] Dmitriev A.A., Pushkova E.N., Melnikova N.V. (2022). Plant genome sequencing: Modern technologies and novel opportunities for breeding. Mol. Biol..

[25] Yu L., Diao S., Zhang G., Yu J., Zhang T., Luo H., Duan A., Wang J., He C., Zhang J. (2022). Genome sequence and population genomics provide insights into chromosomal evolution and phytochemical innovation of *Hippophae rhamnoides*. Plant Biotechnol. J..

[26] Wu Z., Chen H., Pan Y., Feng H., Fang D., Yang J., Wang Y., Yang J., Sahu S.K., Liu J. (2022). Genome of *Hippophae rhamnoides* provides insights into a conserved molecular mechanism in actinorhizal and rhizobial symbioses. New Phytol..

[27] Yang X., Luo S., Yang S., Duoji C., Wang Q., Chen Z., Yang D., Yang T., Wan X., Yang Y. (2024). Chromosome-level genome assembly of *Hippophae rhamnoides* variety. Sci. Data.

[28] Wang R., Wu B., Jian J., Tang Y., Zhang T., Song Z., Zhang W., Qiong L. (2022). How to survive in the world’s third poplar: Insights from the genome of the highest altitude woody plant, *Hippophae tibetana* (Elaeagnaceae). Front. Plant Sci..

[29] Zhang G., Song Y., Chen N., Wei J., Zhang J., He C. (2024). Chromosome-level genome assembly of *Hippophae tibetana* provides insights into high-altitude adaptation and flavonoid biosynthesis. BMC Biol..

[30] Chen M., Yang D., Yang S., Yang X., Chen Z., Yang T., Yang Y., Yang Y. (2024). Chromosome-level genome assembly of *Hippophae gyantsensis*. Sci. Data.

[31] Chen N., Zhang G.Y., Song Y.T., Yang Y., Zhang J.G., He C.Y. (2025). A chromosome-scale genome of *Hippophae neurocarpa* provides new insights into serotonin biosynthesis and chlorophyll-derived brown fruit coloration. Plant J. Cell Mol. Biol..

[32] Cheng H., Qu H., McKenzie S., Lawrence K.R., Windsor R., Vella M., Park P.J., Li H. (2025). Efficient near telomere-to-telomere assembly of Nanopore Simplex reads. bioRxiv.

[33] Lu D., Liu C., Ji W., Xia R., Li S., Liu Y., Liu N., Liu Y., Deng X.W., Li B. (2024). Nanopore ultra-long sequencing and adaptive sampling spur plant complete telomere-to-telomere genome assembly. Mol. Plant.

[34] Zhang T., Li H., Jiang M., Hou H., Gao Y., Li Y., Wang F., Wang J., Peng K., Liu Y.X. (2024). Nanopore sequencing: Flourishing in its teenage years. J. Genet. Genom..

[35] Sigova E.A., Dvorianinova E.M., Arkhipov A.A., Rozhmina T.A., Kudryavtseva L.P., Kaplun A.M., Bodrov Y.V., Pavlova V.A., Borkhert E.V., Zhernova D.A. (2024). Nanopore data-driven T2T genome assemblies of *Colletotrichum lini* strains. J. Fungi.

[36] Arkhipov A.A., Dvorianinova E.M., Turba A.A., Novakovskiy R.O., Zubarev Y.A., Predushchenko P.A., Sigova E.A., Zhernova D.A., Borkhert E.V., Pushkova E.N. (2024). Identification and analysis of *KAS II*, *FAT*, *SAD*, and *FAD* gene families in *Hippophae rhamnoides*. Plants.

[37] Shchapov N.S. (1979). Caryology of *Hippophae rhamnoides* L.. Cytol. Genet..

[38] Rousi A., Arohonka T. (1980). C-bands and ploidy level of *Hippophaë rhamnoides*. Hereditas.

[39] Melnikova N.V., Arkhipov A.A., Zubarev Y.A., Novakovskiy R.O., Turba A.A., Pushkova E.N., Zhernova D.A., Mazina A.S., Dvorianinova E.M., Sigova E.A. (2025). Genetic diversity of *Hippophae rhamnoides* varieties with different fruit characteristics based on whole-genome sequencing. Front. Plant Sci..

[40] Scarano C., Veneruso I., De Simone R.R., Di Bonito G., Secondino A., D’Argenio V. (2024). The third-generation sequencing challenge: Novel insights for the omic sciences. Biomolecules.

[41] Chaushevska M., Alapont-Celaya K., Schack A.K., Krych L., Garrido Navas M.C., Krithara A., Madjarov G. (2025). Get ready for short tandem repeats analysis using long reads-the challenges and the state of the art. Front. Genet..

[42] Xu R., Pan Z., Nakagawa T. (2023). Gross chromosomal rearrangement at centromeres. Biomolecules.

[43] Naish M. (2024). Bridging the gap: Unravelling plant centromeres in the telomere-to-telomere era. New Phytol..

[44] Jones A., Davies H.M., Voelker T.A. (1995). Palmitoyl-acyl carrier protein (ACP) thioesterase and the evolutionary origin of plant acyl-ACP thioesterases. Plant Cell.

[45] Shanklin J., Somerville C. (1991). Stearoyl-acyl-carrier-protein desaturase from higher plants is structurally unrelated to the animal and fungal homologs. Proc. Natl. Acad. Sci. USA.

[46] Okuley J., Lightner J., Feldmann K., Yadav N., Lark E., Browse J. (1994). Arabidopsis *FAD2* gene encodes the enzyme that is essential for polyunsaturated lipid synthesis. Plant Cell.

[47] Arondel V., Lemieux B., Hwang I., Gibson S., Goodman H.M., Somerville C.R. (1992). Map-based cloning of a gene controlling omega-3 fatty acid desaturation in *Arabidopsis*. Science.

[48] Li H., Durbin R. (2024). Genome assembly in the telomere-to-telomere era. Nat. Rev. Genet..

[49] Chen J., Wang Z., Tan K., Huang W., Shi J., Li T., Hu J., Wang K., Wang C., Xin B. (2023). A complete telomere-to-telomere assembly of the maize genome. Nat. Genet..

[50] Chen W., Wang X., Sun J., Wang X., Zhu Z., Ayhan D.H., Yi S., Yan M., Zhang L., Meng T. (2024). Two telomere-to-telomere gapless genomes reveal insights into *Capsicum* evolution and capsaicinoid biosynthesis. Nat. Commun..

[51] Liu W., Xu S., Ou C., Liu X., Zhuang F., Deng X.W. (2024). T2T genomes of carrot and *Alternaria dauci* and their utility for understanding host-pathogen interactions during carrot leaf blight disease. Plant J. Cell Mol. Biol..

[52] Zhang Y., Zhao M., Tan J., Huang M., Chu X., Li Y., Han X., Fang T., Tian Y., Jarret R. (2024). Telomere-to-telomere *Citrullus* super-pangenome provides direction for watermelon breeding. Nat. Genet..

[53] Zhang C., Xie L., Yu H., Wang J., Chen Q., Wang H. (2023). The T2T genome assembly of soybean cultivar ZH13 and its epigenetic landscapes. Mol. Plant.

[54] Li M., Chen C., Wang H., Qin H., Hou S., Yang X., Jian J., Gao P., Liu M., Mu Z. (2024). Telomere-to-telomere genome assembly of sorghum. Sci. Data.

[55] Yan H., Han J., Jin S., Han Z., Si Z., Yan S., Xuan L., Yu G., Guan X., Fang L. (2025). Post-polyploidization centromere evolution in cotton. Nat. Genet..

[56] Zelenka T., Spilianakis C. (2021). HiChIP and Hi-C protocol optimized for primary murine T cells. Methods Protoc..

[57] Pushkova E.N., Povkhova L.V., Dvorianinova E.M., Novakovskiy R.O., Rozhmina T.A., Gryzunov A.A., Sigova E.A., Zhernova D.A., Borkhert E.V., Turba A.A. (2024). Expression of *FAD* and *SAD* genes in developing seeds of flax varieties under different growth conditions. Plants.

[58] De Coster W., Rademakers R. (2023). NanoPack2: Population-scale evaluation of long-read sequencing data. Bioinformatics.

[59] Gurevich A., Saveliev V., Vyahhi N., Tesler G. (2013). QUAST: Quality assessment tool for genome assemblies. Bioinformatics.

[60] Manni M., Berkeley M.R., Seppey M., Simao F.A., Zdobnov E.M. (2021). BUSCO update: Novel and streamlined workflows along with broader and deeper phylogenetic coverage for scoring of eukaryotic, prokaryotic, and viral genomes. Mol. Biol. Evol..

[61] Kielbasa S.M., Wan R., Sato K., Horton P., Frith M.C. (2011). Adaptive seeds tame genomic sequence comparison. Genome Res..

[62] Brown M.R., Manuel Gonzalez de La Rosa P., Blaxter M. (2025). tidk: A toolkit to rapidly identify telomeric repeats from genomic datasets. Bioinformatics.

[63] The Matplotlib Development Team (2025). Matplotlib: Visualization with Python. Zenodo. https://zenodo.org/records/20149089.

[64] Servant N., Varoquaux N., Lajoie B.R., Viara E., Chen C.J., Vert J.P., Heard E., Dekker J., Barillot E. (2015). HiC-Pro: An optimized and flexible pipeline for Hi-C data processing. Genome Biol..

[65] Durand N.C., Shamim M.S., Machol I., Rao S.S., Huntley M.H., Lander E.S., Aiden E.L. (2016). Juicer provides a one-click system for analyzing loop-resolution Hi-C experiments. Cell Syst..

[66] Chen S., Zhou Y., Chen Y., Gu J. (2018). fastp: An ultra-fast all-in-one FASTQ preprocessor. Bioinformatics.

[67] Langmead B., Salzberg S.L. (2012). Fast gapped-read alignment with Bowtie 2. Nat. Methods.

[68] Marcais G., Kingsford C. (2011). A fast, lock-free approach for efficient parallel counting of occurrences of k-mers. Bioinformatics.

[69] Ranallo-Benavidez T.R., Jaron K.S., Schatz M.C. (2020). GenomeScope 2.0 and Smudgeplot for reference-free profiling of polyploid genomes. Nat. Commun..

[70] Rhie A., Walenz B.P., Koren S., Phillippy A.M. (2020). Merqury: Reference-free quality, completeness, and phasing assessment for genome assemblies. Genome Biol..

[71] Flynn J.M., Hubley R., Goubert C., Rosen J., Clark A.G., Feschotte C., Smit A.F. (2020). RepeatModeler2 for automated genomic discovery of transposable element families. Proc. Natl. Acad. Sci. USA.

[72] Storer J., Hubley R., Rosen J., Wheeler T.J., Smit A.F. (2021). The Dfam community resource of transposable element families, sequence models, and genome annotations. Mob. DNA.

[73] Bao W., Kojima K.K., Kohany O. (2015). Repbase Update, a database of repetitive elements in eukaryotic genomes. Mob. DNA.

[74] Gabriel L., Bruna T., Hoff K.J., Ebel M., Lomsadze A., Borodovsky M., Stanke M. (2024). BRAKER3: Fully automated genome annotation using RNA-seq and protein evidence with GeneMark-ETP, AUGUSTUS, and TSEBRA. Genome Res..

[75] Kuznetsov D., Tegenfeldt F., Manni M., Seppey M., Berkeley M., Kriventseva E.V., Zdobnov E.M. (2023). OrthoDB v11: Annotation of orthologs in the widest sampling of organismal diversity. Nucleic Acids Res..

[76] Krzywinski M., Schein J., Birol I., Connors J., Gascoyne R., Horsman D., Jones S.J., Marra M.A. (2009). Circos: An information aesthetic for comparative genomics. Genome Res..

[77] Wang Y., Tang H., Debarry J.D., Tan X., Li J., Wang X., Lee T.H., Jin H., Marler B., Guo H. (2012). MCScanX: A toolkit for detection and evolutionary analysis of gene synteny and collinearity. Nucleic Acids Res..

[78] Mistry J., Chuguransky S., Williams L., Qureshi M., Salazar G.A., Sonnhammer E.L.L., Tosatto S.C.E., Paladin L., Raj S., Richardson L.J. (2021). Pfam: The protein families database in 2021. Nucleic Acids Res..

[79] Wickham H. (2016). ggplot2: Elegant Graphics for Data Analysis.

[80] Letunic I., Bork P. (2024). Interactive Tree of Life (iTOL) v6: Recent updates to the phylogenetic tree display and annotation tool. Nucleic Acids Res..

[81] Krasnov G.S., Dmitriev A.A., Kudryavtseva A.V., Shargunov A.V., Karpov D.S., Uroshlev L.A., Melnikova N.V., Blinov V.M., Poverennaya E.V., Archakov A.I. (2015). PPLine: An automated pipeline for SNP, SAP, and splice variant detection in the context of proteogenomics. J. Proteome Res..

[82] Robinson J.T., Thorvaldsdottir H., Winckler W., Guttman M., Lander E.S., Getz G., Mesirov J.P. (2011). Integrative genomics viewer. Nat. Biotechnol..

