# Supplementary material for: Nanopore Data-Driven Near-T2T Genome Assembly of Hippophae rhamnoides ssp. mongolica Rousi and Its Complex Annotation
Source: Plants (Basel). 2026 Jun 2;15(11):1726. doi: 10.3390/plants15111726 (PMC13259092; doi:10.3390/plants15111726)
Supplement: Supplementary file 1 [file plants-15-01726-s001.zip › Supplementary Figure S1_2026.04.26.pdf]

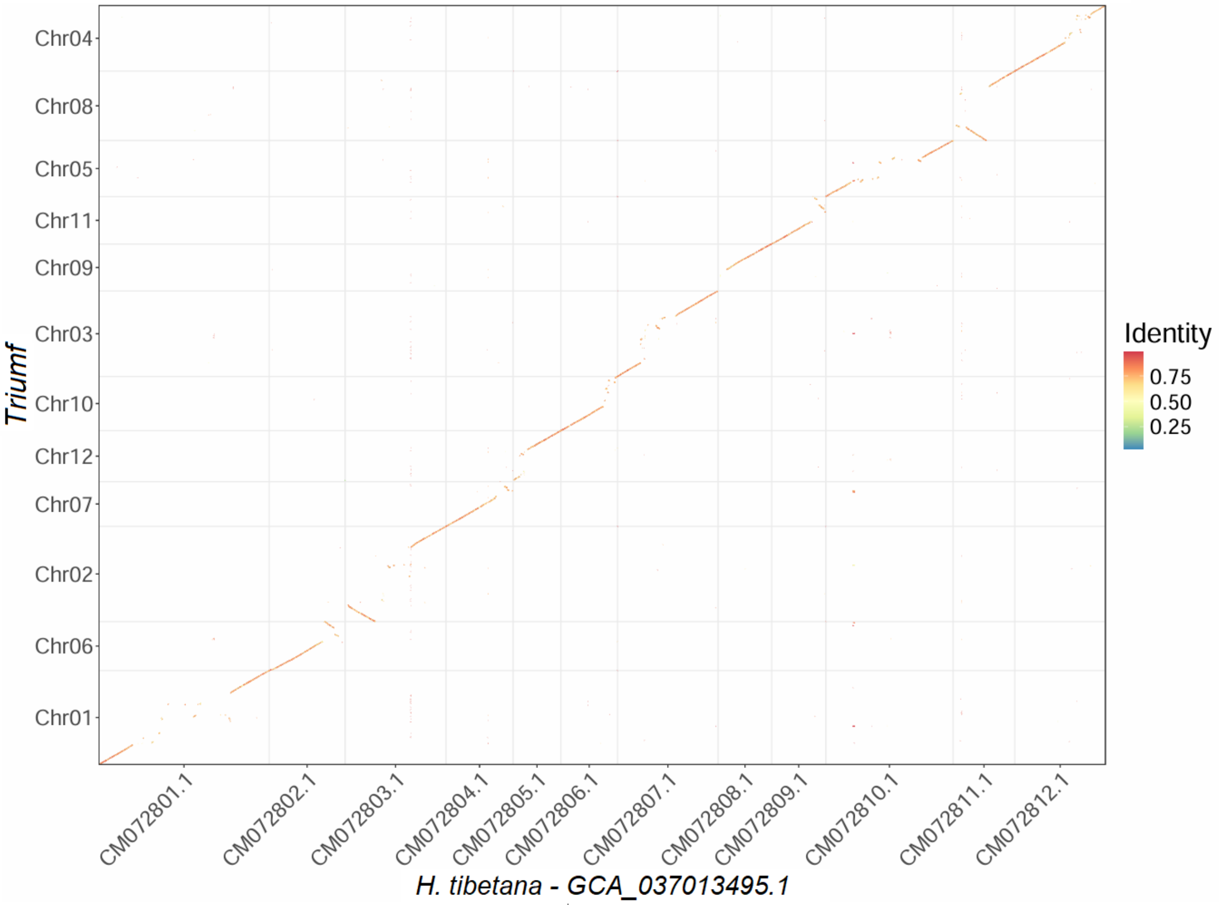

Supplementary Figure S1. Global alignment of the genome assemblies of *H. rhamnoides* variety Triumf and other *Hippophae* species. (**a**) variety Triumf (Y axis) and *H. tibetana* [29], NCBI, GCA_037013495.1 (X axis).

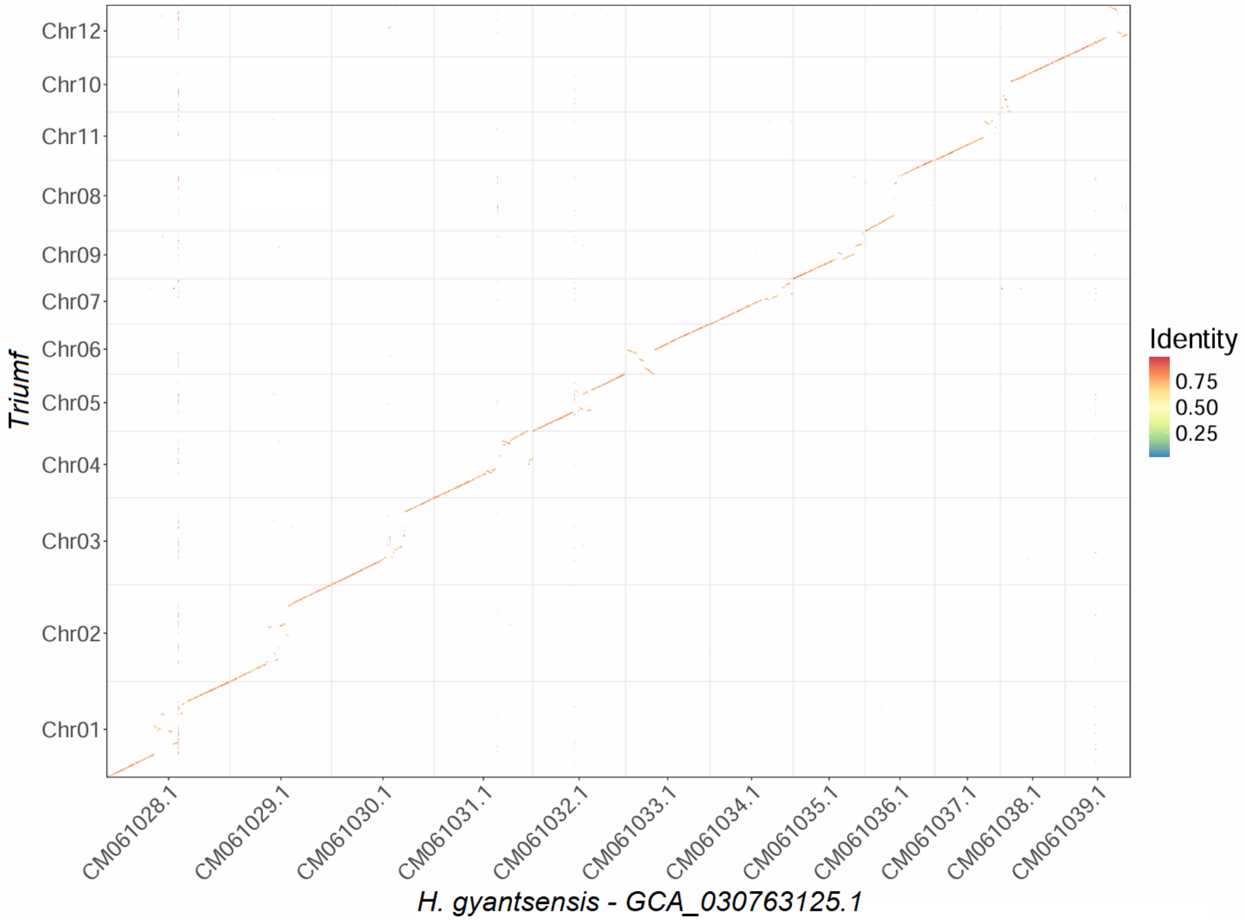

Supplementary Figure S1. Global alignment of the genome assemblies of *H. rhamnoides* variety Triumf and other *Hippophae* species. (**b**) variety Triumf (Y axis) and *H. gyantsensis* [30], NCBI, GCA_030763125.1 (X axis).
