# Supplementary material for: Nanopore Data-Driven Near-T2T Genome Assembly of Hippophae rhamnoides ssp. mongolica Rousi and Its Complex Annotation
Source: Plants (Basel). 2026 Jun 2;15(11):1726. doi: 10.3390/plants15111726 (PMC13259092; doi:10.3390/plants15111726)
Supplement: Supplementary file 1 [file plants-15-01726-s001.zip › Supplementary Figure S2_2026.05.16.pdf]

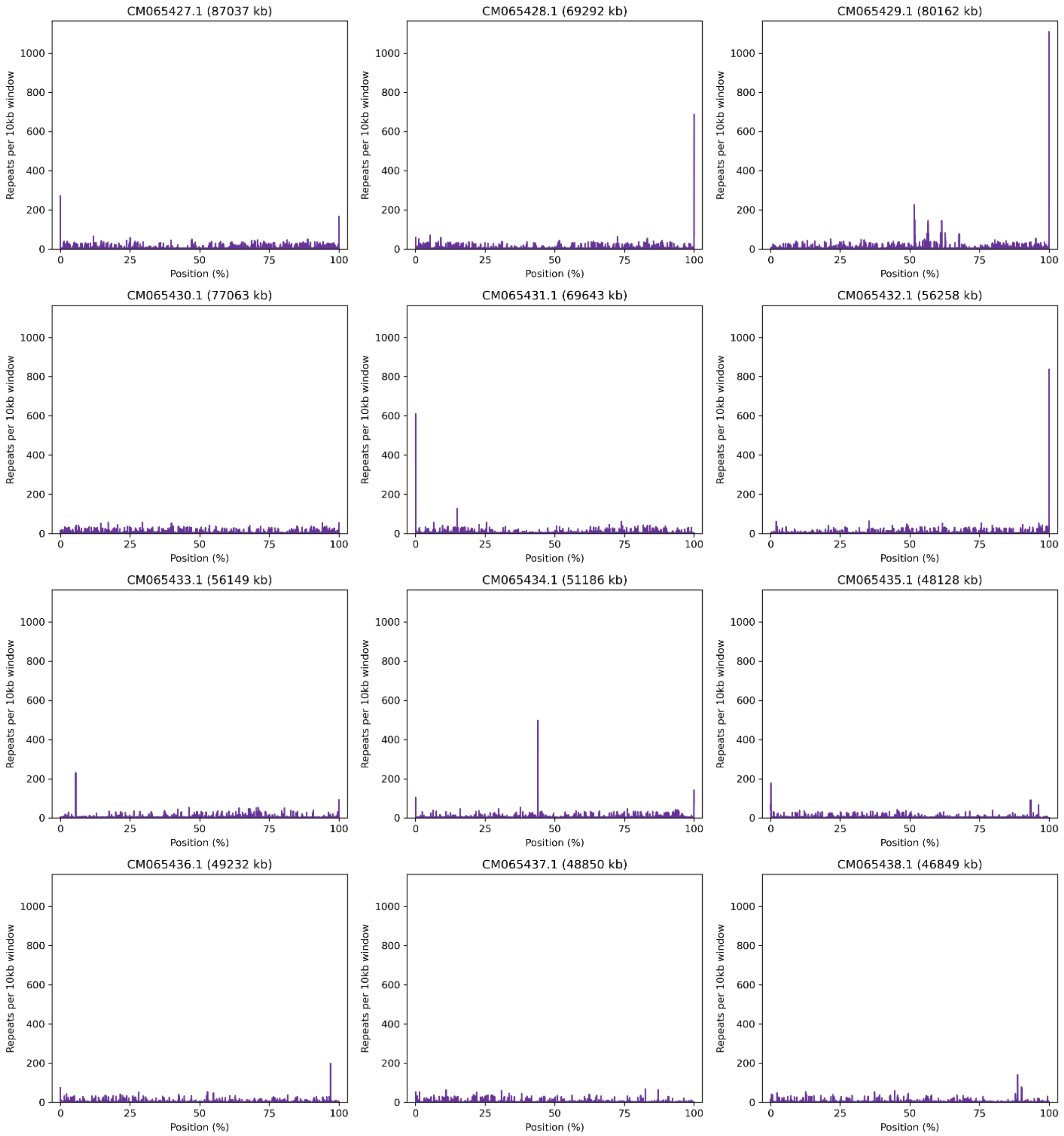

Supplementary Figure S2. Density of telomeric repeats in the analyzed genome assemblies of *Hippophae* species. (**a**) *H. rhamnoides* ssp. *mongolica* × *H. rhamnoides* ssp. *sinensis*, NCBI, GCA_033030585.1. Chromosome size is indicated for each chromosome in brackets.

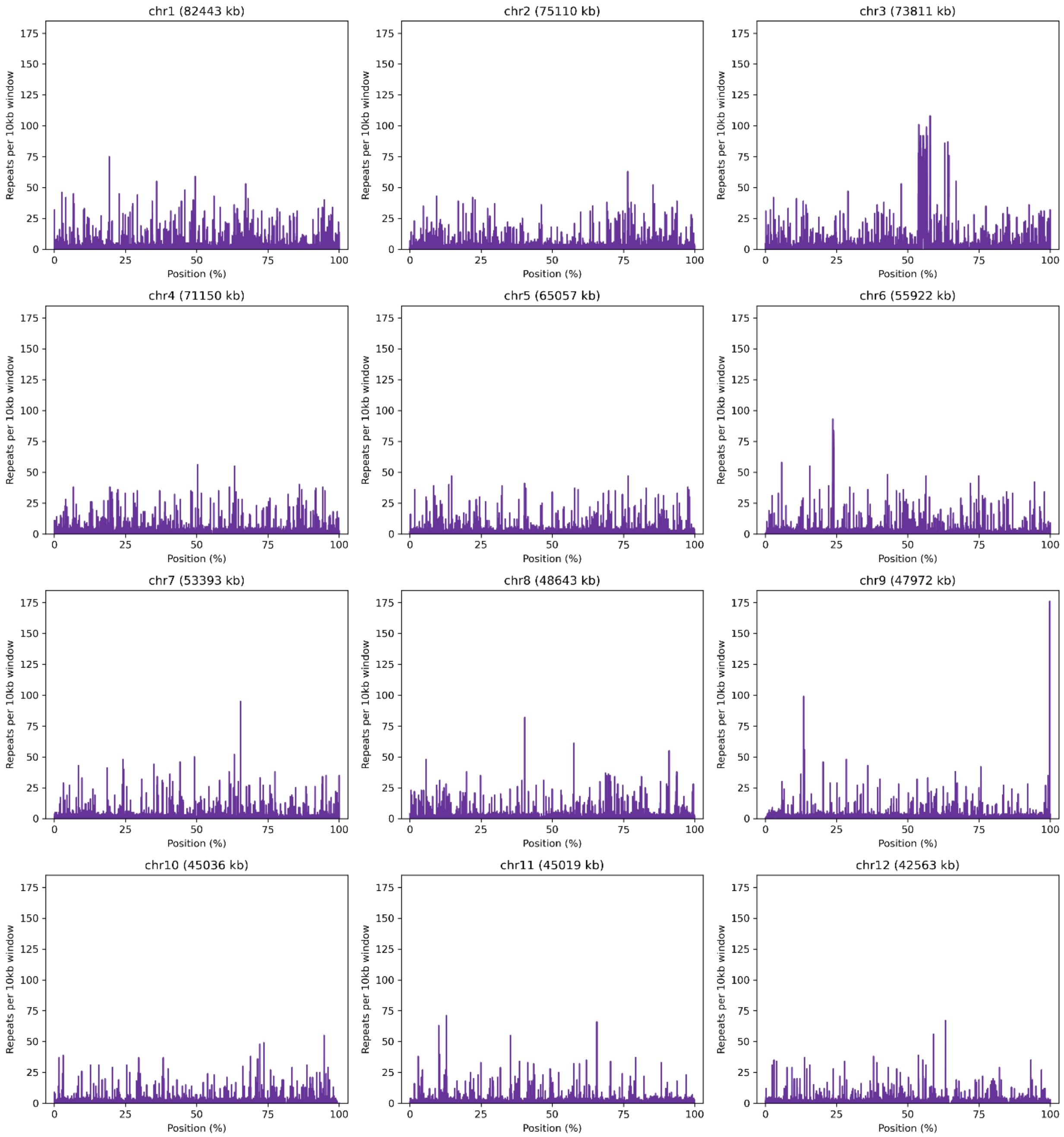

Supplementary Figure S2. Density of telomeric repeats in the analyzed genome assemblies of *Hippophae* species. (**b**) *H. rhamnoides*, CNGB, CNA0022752. Chromosome size is indicated for each chromosome in brackets.

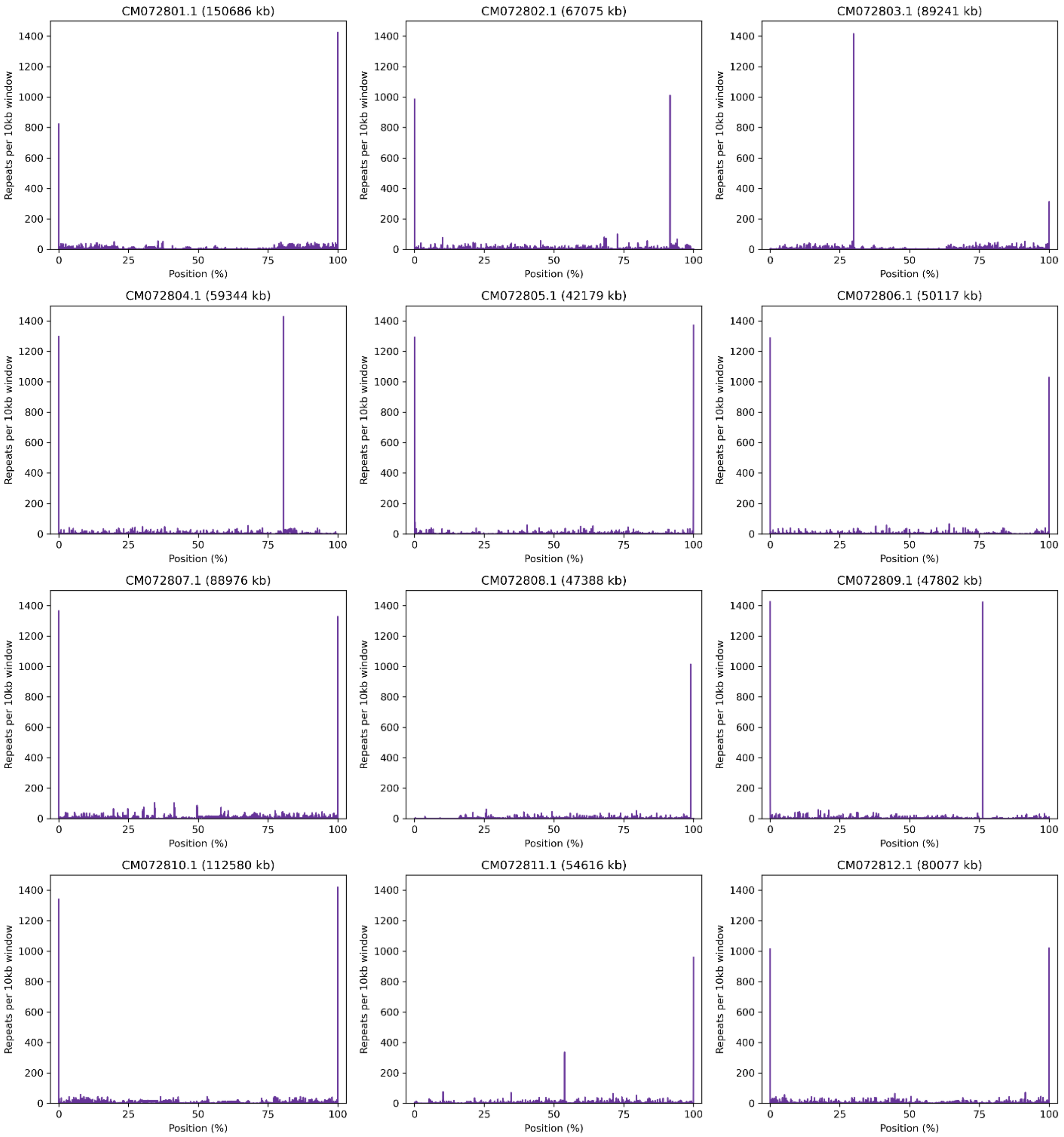

Supplementary Figure S2. Density of telomeric repeats in the analyzed genome assemblies of *Hippophae* species. (**c**) *H. tibetana*, NCBI, GCA_037013495.1. Chromosome size is indicated for each chromosome in brackets.

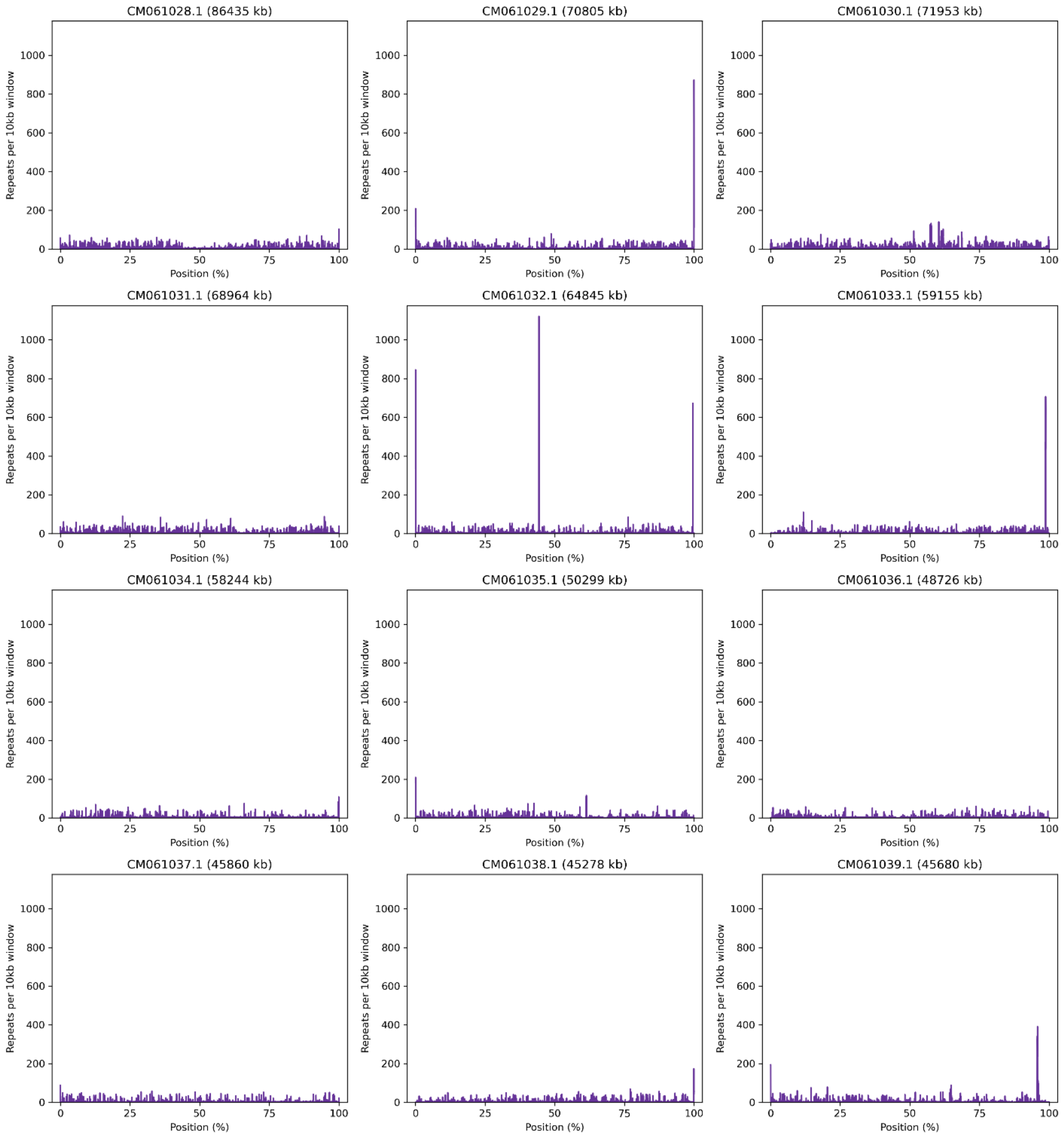

Supplementary Figure S2. Density of telomeric repeats in the analyzed genome assemblies of *Hippophae* species. (**d**) *H. gyantsensis*, NCBI, GCA_030763125.1. Chromosome size is indicated for each chromosome in brackets.
