# Supplementary material for: Nanopore Data-Driven Near-T2T Genome Assembly of Hippophae rhamnoides ssp. mongolica Rousi and Its Complex Annotation
Source: Plants (Basel). 2026 Jun 2;15(11):1726. doi: 10.3390/plants15111726 (PMC13259092; doi:10.3390/plants15111726)
Supplement: Supplementary file 1 [file plants-15-01726-s001.zip › Supplementary Figure S3_2026.04.26.pdf]

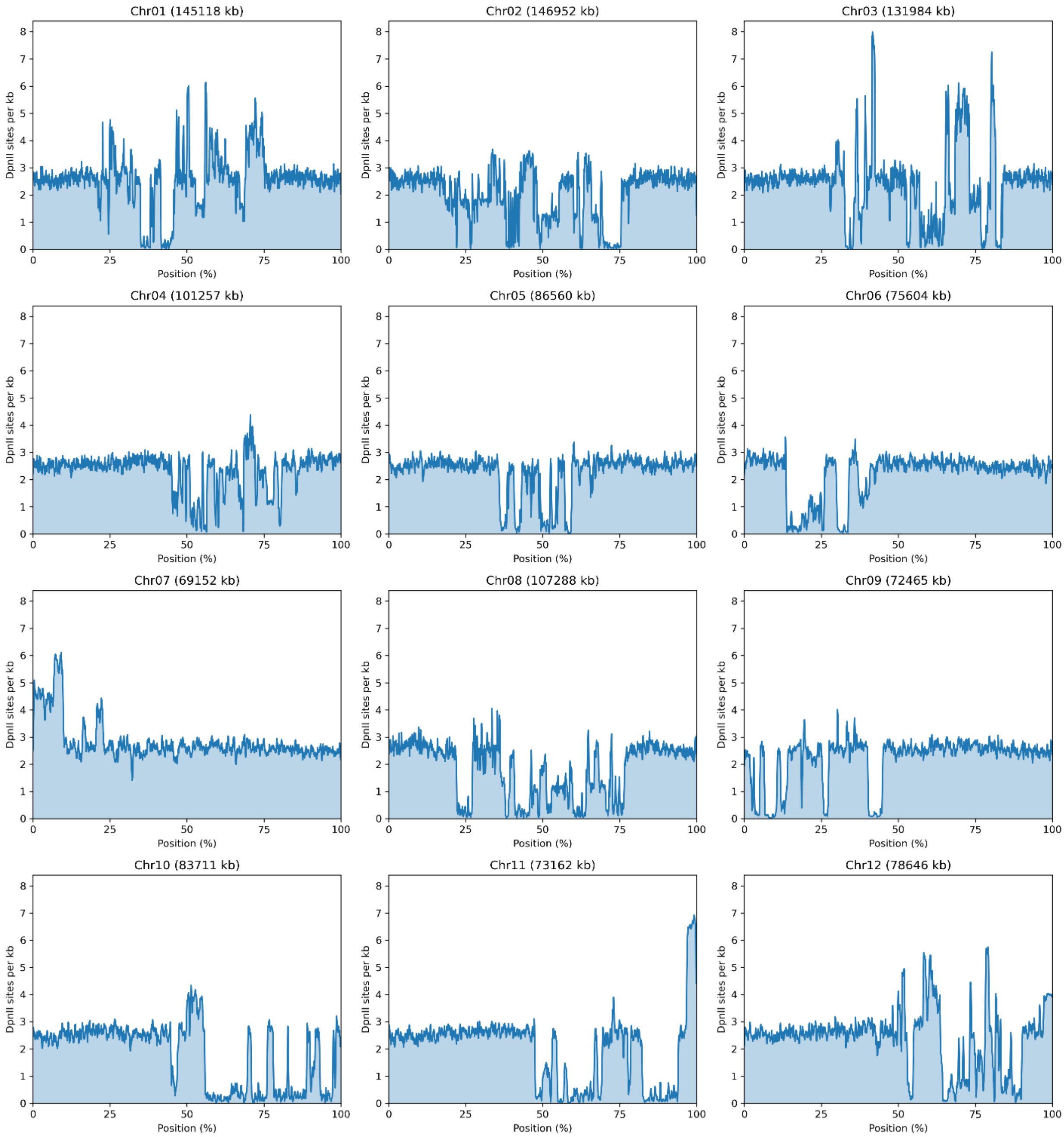

Supplementary Figure S3. Density of restriction site for the DpnII enzyme (GATC) in the *H. rhamnoides* variety Triumf genome assembly. Chromosome size is indicated for each chromosome in brackets.
