# Supplementary material for: Nanopore Data-Driven Near-T2T Genome Assembly of Hippophae rhamnoides ssp. mongolica Rousi and Its Complex Annotation
Source: Plants (Basel). 2026 Jun 2;15(11):1726. doi: 10.3390/plants15111726 (PMC13259092; doi:10.3390/plants15111726)
Supplement: Supplementary file 1 [file plants-15-01726-s001.zip › Supplementary Figure S4_2026.04.26.pdf]

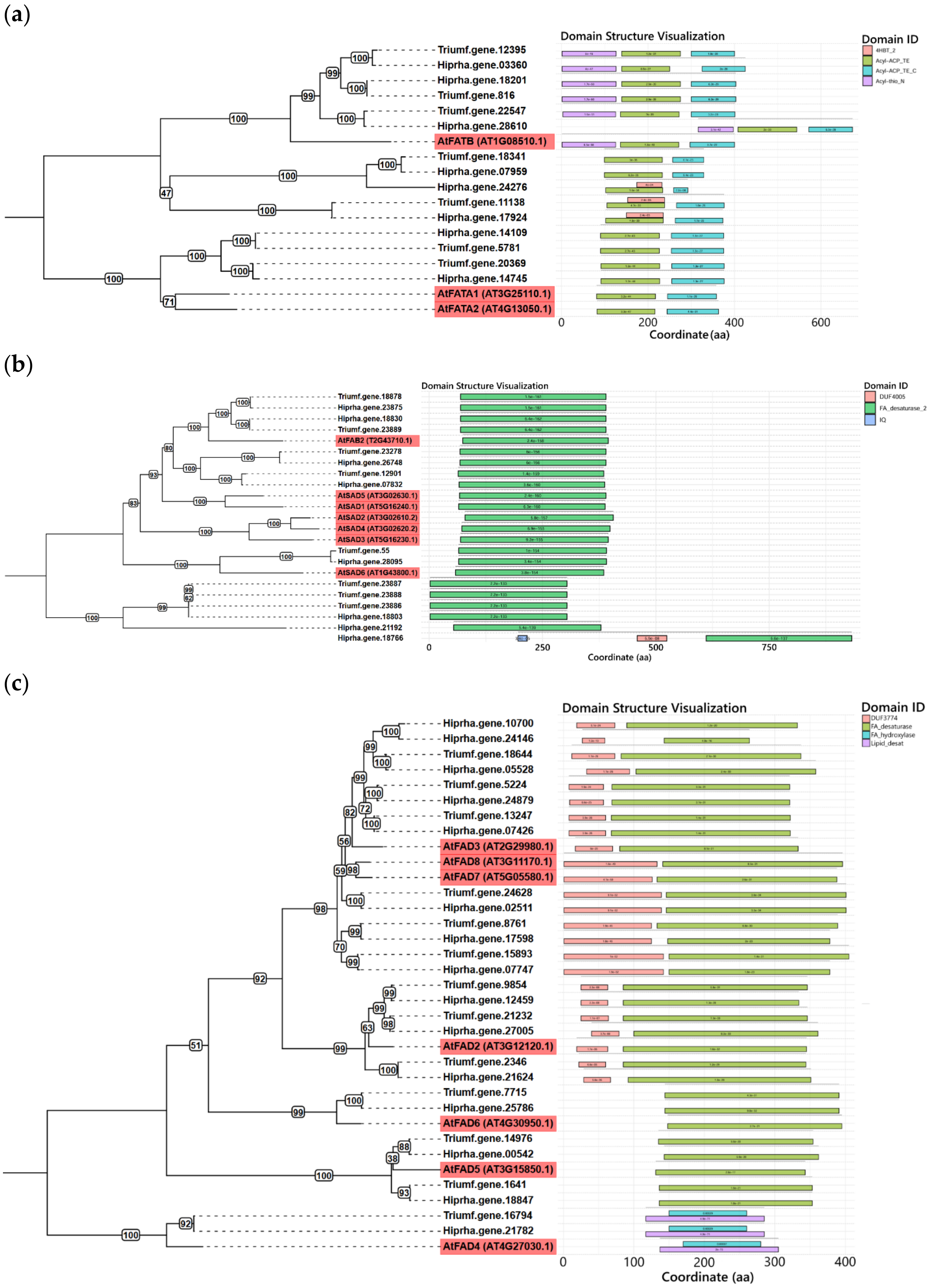

Supplementary Figure S4. Conserved domain structure of the FAT, SAD, and FAD proteins identified in the *H. rhamnoides* genome assemblies of variety Triumf and genotype CNA0022752. (**a**) FATA and FATB proteins, (**b**) SAD proteins, (**c**) FAD proteins.

Conserved domains were identified using the Pfam database. The proteins of the reference species *Arabidopsis thaliana* (At…) and their homologs identified in two annotated genome assemblies of *H. rhamnoides* are shown: Hiprha.gene.xxx – the CNA0022752 genome assembly (CNGB), Triumf.gene.xxx – the Triumf genome assembly obtained in the present study.
