# Supplementary material for: Nanopore Data-Driven Near-T2T Genome Assembly of Hippophae rhamnoides ssp. mongolica Rousi and Its Complex Annotation
Source: Plants (Basel). 2026 Jun 2;15(11):1726. doi: 10.3390/plants15111726 (PMC13259092; doi:10.3390/plants15111726)
Supplement: Supplementary file 1 [file plants-15-01726-s001.zip › Supplementary Figure S6_2026.04.26.pdf]

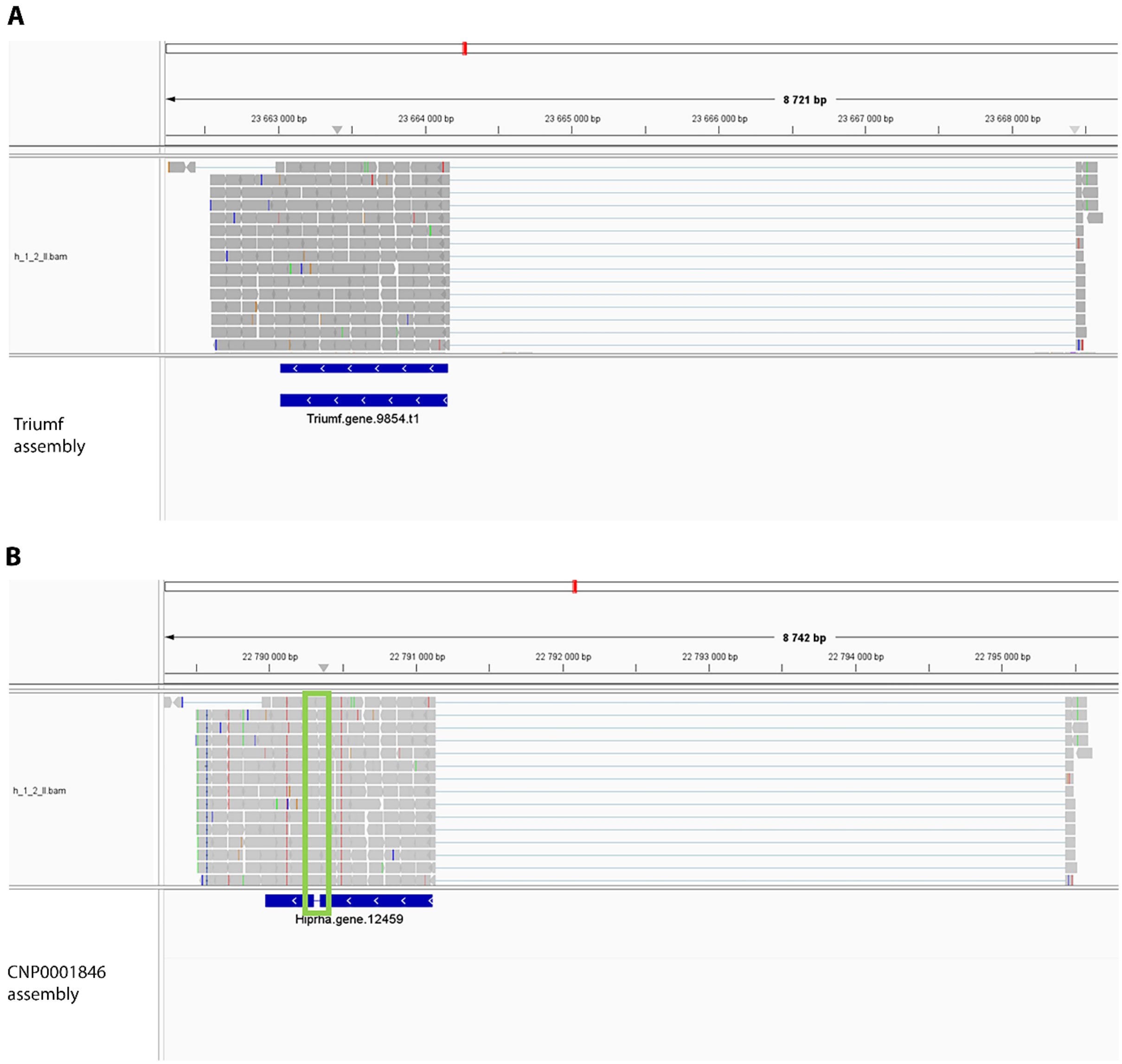

Supplementary Figure S6. Mapping of RNA-Seq reads to the *H. rhamnoides* Triumf and CNA0022752 (CNGB, Project ID CNP0001846) genome assemblies for the *FAD2* gene region: (**a**) *Triumf.gene.9854* in the Triumf assembly and (**b**) *Hiprha.gene.12459* in the CNA0022752 assembly.
