# Supplementary material for: Nanopore Data-Driven Near-T2T Genome Assembly of Hippophae rhamnoides ssp. mongolica Rousi and Its Complex Annotation
Source: Plants (Basel). 2026 Jun 2;15(11):1726. doi: 10.3390/plants15111726 (PMC13259092; doi:10.3390/plants15111726)
Supplement: Supplementary file 1 [file plants-15-01726-s001.zip › Supplementary Figure S8_2026.05.16.pdf]

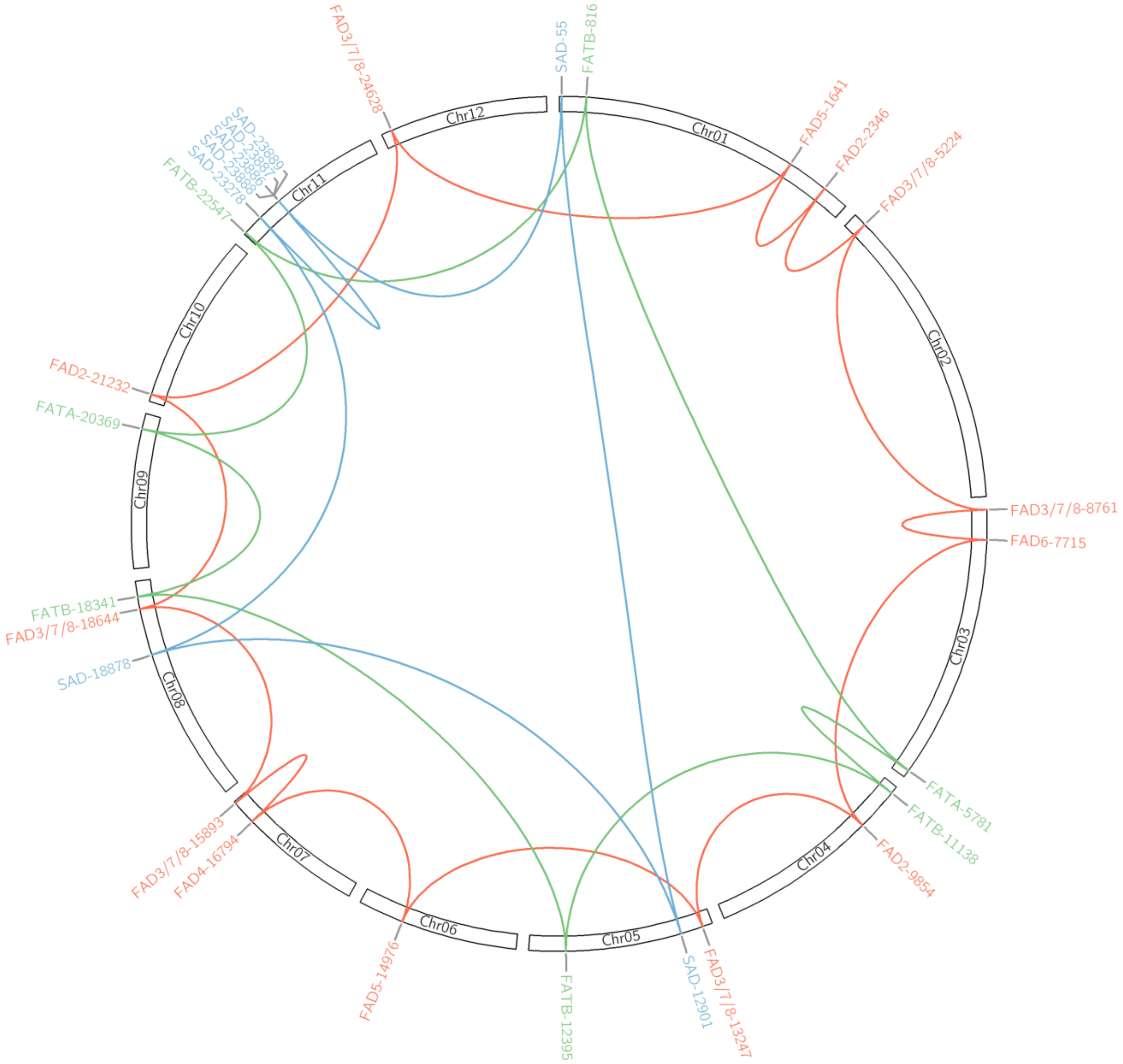

Supplementary Figure S8. Location of identified in the present study *FAT*, *SAD*, and *FAD* genes on twelve chromosomes of the sea buckthorn variety Triumf genome assembly. Gene names "*Triumf.gene.xxx*" are presented as "gene family-xxx" (e.g., *Triumf.gene.55* is presented as SAD-55).
